# A Novel and Effective Chromatographic Approach to the Separation of Isoflavone Derivatives from *Pueraria lobata*

**DOI:** 10.3390/molecules20034238

**Published:** 2015-03-05

**Authors:** Jiang Fu, Wenguang Jing, Weihao Wang, Sha Chen, Jun Zhang, An Liu

**Affiliations:** 1 Institute of Chinese Materia Medica, China Academy of Chinese Medical Sciences, Beijing 100700, China; E-Mails: fujiangdali@163.com (J.F.); 163webpasser@163.com (W.J.); olive_wh@126.com (W.W.); sand1107@163.com (S.C.); junzhang654321@163.com (J.Z.); 2 College of Pharmacy, Jiang Xi University of Traditional Chinese Medicine, Nanchang 330006, China

**Keywords:** flash chromatography, preparative high performance liquid chromatography, online mode separation, offline mode separation, *Pueraria lobata*

## Abstract

A novel and effective chromatographic approach to the separation and purification of isoflavone compounds from *Pueraria lobata* is described. The method is based on flash chromatography (FC), coupled to preparative high performance liquid chromatography (prep-HPLC) via a six-way valve. The FC step comprised tandem reversed phase columns, pre-packed with MCI gel (Mitsubishi Chemical Corp., Tokyo, Japan) and C18 (Fuji Silysia Chemical Ltd, Osaka, Japan) resin, respectively, and was designed to separate a crude *Pueraria lobata* extract into several preliminary fractions. Fractions containing the target compounds were then directly injected via the six-way valve into prep-HPLC columns, without further treatment, for final isolation and purification. Nine isoflavonoids were successfully isolated, three through an online mode and the other six through an offline mode. The purities of all compounds exceeded 95.0%, as determined by HPLC with an UV-vis photodiode array detector. The convenience, low solvent consumption, and time-saving advantages of this method offer an attractive and promising approach to the isolation of natural products.

## 1. Introduction

The root of *Pueraria lobata* (Wild.) Ohwi is an important Chinese herb used in China as an herbal medicine, named “Ge-Gen”, to treat hypertension and anginapectoris [1]. Modern pharmacological studies have shown that it possesses antioxidant [2,3], anti-boneloss [4], antihypertensive [5], antidepressant [6], anticancer [7,8] and estrogen-like effects [9,10]. It also has been used as an analgesic and muscle relaxant [11] and to treat alcohol dependence [12]. In recent years, the literature has reported isoflavones as the major active compounds contained in the crude *Pueraria lobata* drug and its preparations [13,14,15,16,17,18].

Conventional methods have been used to separate and identify isoflavones from *Pueraria lobata*, such as solvent extraction, crystallization and avariety of chromatographic methods. Previous reports state that high-speed countercurrent chromatography (HSCCC) technology can effectively separate and identify isoflavones [19]. However, there are some disadvantages to the above-mentioned methods. First, to achieve primary separation and enrichment of the target components via conventional methods, repeated chromatographic processing of the crude extract is required, which uses large volumes of organic solvents and is greatly time consuming. Secondly, complicated (multi-step) procedures are generally accompanied by poor sample recoveries. Finally, HSCCC requires extensive preparatory work, such as preliminary screening and optimization of the solvent system, based on determining the partition coefficient (*K*) of the target compounds, and has poor sensitivity and separation efficiency, especially for trace constituents.

**Figure 1:**
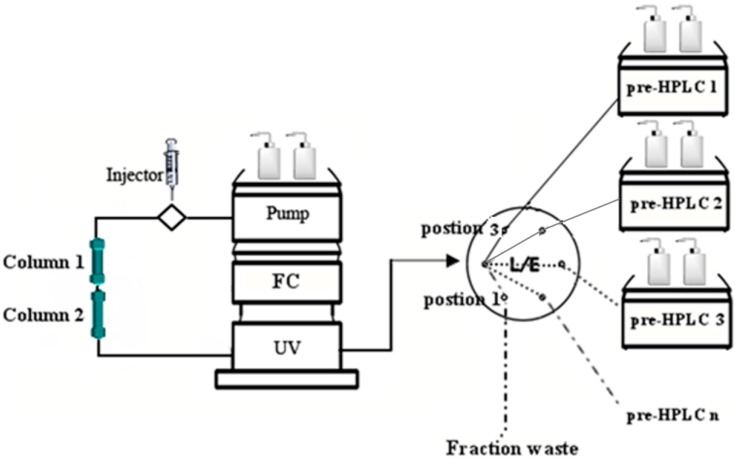
Diagram of flash chromatography coupled with multiple preparative high performance liquid chromatography. FC (flash chromatography).

Flash chromatography (FC) offers rapid separation and low solvent consumption and is a reliable pretreatment process that has been applied extensively to the separation and purification of monomeric substances. In addition, preparative high performance liquid chromatography (prep-HPLC) is a powerful tool that has excellent efficiency and high recovery, especially for the fine separation of trace constituents. In the present study, we successfully identified a combined method to solve the above-mentioned difficulties. We used FC, coupled to two sets of prep-HPLC, connected via a six-way valve (Figure 1). With this combined separation approach, nine isoflavones of *Pueraria lobata* were isolated and purified, namely 3'-hydroxypuerarin-6''-*O*-glucoside (**1**), puerarin-6''-*O*-glucoside (**2**), daidzein-4',7-*O*-diglucoside (**3**), 3'-hydroxypuerarin (**4**), 3'-hydroxypuerarin-6''-*O*-apioside (**5**), puerarin (**6**), 3'-methoxypuerarin (**7**), daidzein8-*C*-apiosyl(1→6) glucoside (**8**) and daidzin (**9**). Our study shows that FC coupled with prep-HPLC has the following distinct advantages over other methods: rapid separation, high recovery, low solvent consumption and elimination of the need for pretreatment.

This is the first report of the separation and purification of isoflavones from *Pueraria lobata* by a coupled method, and this is the first time that FC coupled with prep-HPLC has been described as a powerful method in separation and purification technology.

## 2. Results and Discussion

### 2.1. FC Conditions and Tandem Chromatographic Columns

To ensure that the eluted fractions from FC could be loaded directly into the subsequent prep-HPLC steps, the use of a compatible solvent elution system was suggested, to simplify sample pretreatment. In consistency in the mobile phase between FC and prep-HPLC may cause baseline drift, leading to a decline in the separation effect. Therefore, for compatibility with the octadecylsilane (C18) columns used in prep-HPLC, reversed phase chromatographic columns were needed for the FC steps. However, C18 has a low loading capacity. MCI gel (Mitsubishi Chemical Corp.), has the same reversed phase characteristics as C18 resin and possesses a suitable chemical stability, but has a higher sample loading capacity. As a result, a column pre-packed with MCI gel, combined with a column packed with C18, were used in a tandem configuration to combine the strengths and advantages of the two chromatographic beds.

Several solvent systems, including methanol-water, acetonitrile-water and ethanol-water, were tried at different ratios. HPLC analyses of crude samples indicated that the optimal solvent system was methanol-water at different ratios. The flow rate during FC was another critical factor for achieving acceptable separation efficiency. A series of flow rates (5.0, 6.0, 8.0 and 10.0 mL/min) were tested to optimize the separation conditions, and we finally selected 6.0 mL/min as the optimal flow rate.

**Figure 2:**
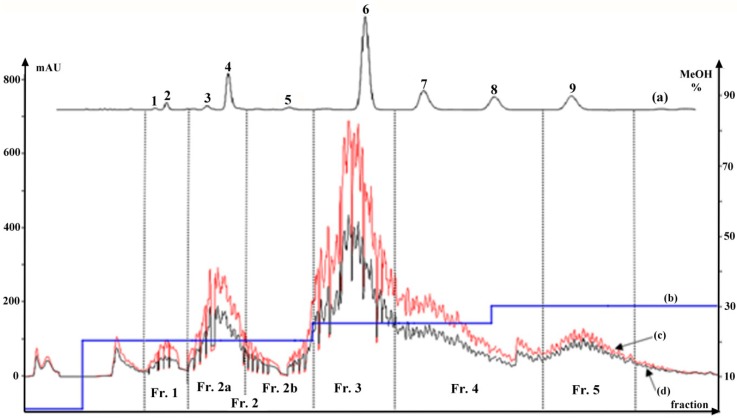
HPLC chromatogram of a crude extract from *Pueraria lobata* (a); gradient elution curve from FC (b); FC chromatogram of crude extract at 250 nm (c) and at 270 nm (d); Compounds **1**–**9**; Fractions 1–5. (Fr., fraction.)

The crude sample solution was manually loaded onto the MCI gel column to conduct preliminary fractionation. After water-soluble impurities were discarded by washing with water at a three-times volume of the column (30 mL), the pre-packed C18 column was cascaded with the MCI gel column, and a stepwise gradient elution was developed to provide an optimal separation. FC chromatograms are shown in Figure 2. All elution shown in the FC chromatogram could be fractionated into five parts (*i.e.*, Fractions 1–5) according to the separation of the target compounds, after which the eluted Fraction 2 could be further divided into two parts, Fraction 2a and Fraction 2b.

### 2.2. Separation Conditions and Procedure for Prep-HPLC

#### 2.2.1. Separation Conditions for Prep-HPLC

The results indicated that a better resolution could be obtained by the addition of formic acid to the mobile phase. Therefore, the mobile phase composition was created using a methanol-acetic acid aqueous solution, with isocratic elution. A flow rate of 9.0 mL/min was used as the optimal condition, and the detection wavelength was set at 250 nm.

#### 2.2.2. Online Mode Separation of Compounds **4**, **6** and **9**

**Figure 3:**
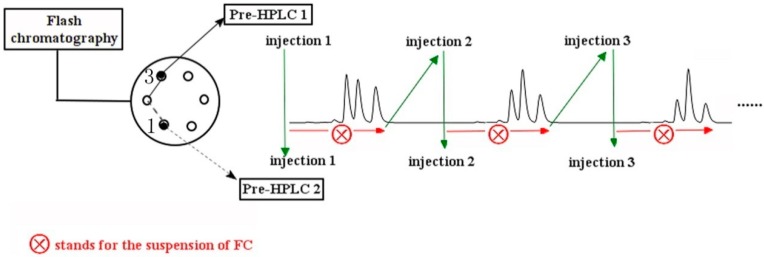
Scheme showing intermittent sample injection performed during offline mode separation. After injection 1 on the prep-HPLC 2 was complete, the valve was switched back to position 1. The second injection (injection 2) was carried out after the loop was fully filled with the elutions, to eliminate peak overlap caused by the short injection intervals.

**Figure 4:**
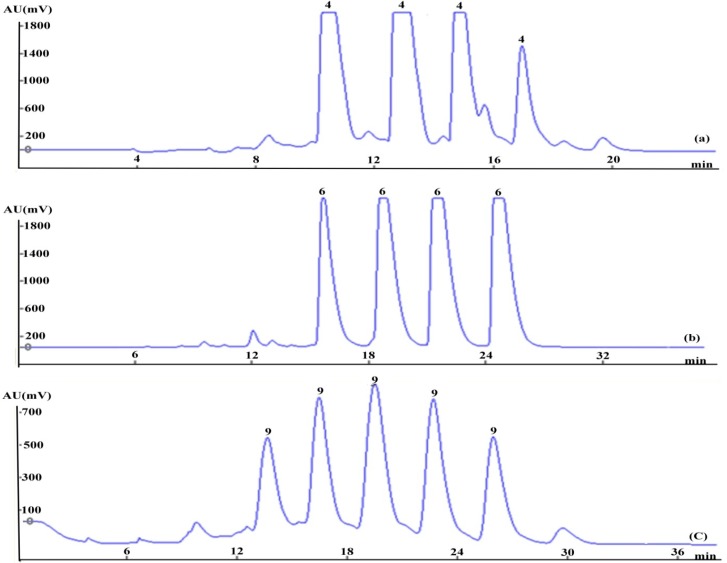
Prep-HPLC chromatograms of **4** (**a**), **6** (**b**) and **9** (**c**), based on online mode separation.

Following the concept of online separation, Fraction 2a, Fraction 3 and Fraction 5, displayed in Figure 3, could be loaded consecutively into the prep-HPLC, without pretreatment. The chromatograms for these fractions separated by prep-HPLC are shown clearly in Figure 4. Fraction 2a, which contains a significant quantity of **4**, was carried out by prep-HPLC (Figure 4a) to yield 20.53 mg of **4** at a high purity of 98.0% and then eluted from the column using 25% methanol to obtain Fraction 3 and Fraction 4. Puerarin (**6**), which is the major component, approximately 4%, of the crude drug, was predominantly distributed in Fraction 3.Continuous injections of Fraction 3 resulted in several consecutive flat peaks of **6** being clearly observed and yielded 85.35 mg at a high purity of 99.8%, as shown in Figure 4b. To minimize elution time, Fraction 5 was eluted by increasing the mobile phase ratio to 70:30 (water:methanol) and separated by prep-HPLC using the same isocratic elution to afford **9** (11.20 mg) at a purity of 95.5% (Figure 4c). Although consecutive injections of the above fractions (Fraction 2a, Fraction 3 and Fraction 5) were performed on prep-HPLC, base line separation between neighboring injections was successfully achieved, and three relatively pure isoflavones (**4**, **6** and **9**) were obtained at purities above 95.0%.

#### 2.2.3. Offline Mode Separation of Compounds **1**, **2**, **3**, **5**, **7** and **8**

**Figure 5:**
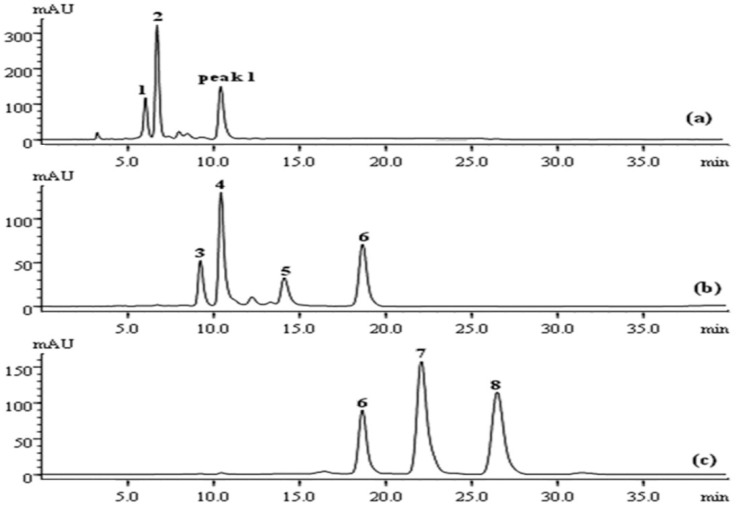
HPLC analyses of the residual elutions: Fraction 1 (**a**); Fraction 2b (**b**); remainder of Fraction 4 (**c**).

Separation of further compounds required us to resolve the critical problem of the difficulty of separating low-content compounds. For example, the concealed peak for **3** in the FC chromatogram is directly related to its low content, and this is related also to the broader peak obtained for **4**, caused by the longer elution time during FC, resulting in difficult separation by prep-HPLC. In spite of the peaks corresponding to **3** observed in Fraction 2b by HPLC analyses (Figure 5b), continuous injection of this fraction into the prep-HPLC could not be performed, because the peaks after two, closely-spaced consecutive injections might overlap. Therefore, the number of prep-HPLC steps combined with FC was limited during the experiment. Therefore, discrete injections between the two sets of prep-HPLC columns, shown in Figure 3, were designed and used. Sample injection was carried out immediately after the loop was filled with the elution; at the same time, the FC was manually paused to await complete separation of the injected sample. When the chromatographic peak from the injection onto the prep-HPLC column was detected, FC was restarted and the operations repeated to perform the subsequent injection. In this way, overlapping of the chromatographic peaks between two consecutive injections could be avoided, with excellent separation, and low-content compounds could clearly be isolated. Intermittent sample injection was certainly effective for the separation of low-content compounds from the crude extract and was equivalent to an offline mode.

**Figure 6:**
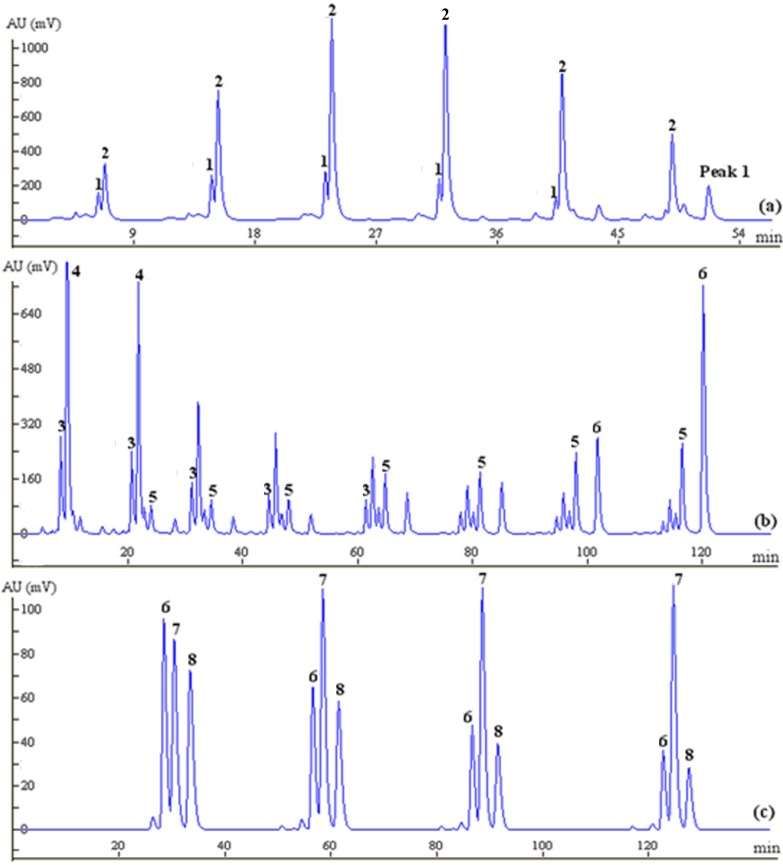
Prep-HPLC chromatograms of **1** and **2** (**a**), **3** and **5** (**b**) and **7** and **8** (**c**) based on offline separation; Peak1shown in (a) was identified as **4** by HPLC analysis.

A similar concealed peak corresponding to **5**, which was at a low concentration in the crude extract, also occurred in the FC chromatogram, and it was later revealed when the Fraction 2b part was subjected to prep-HPLC. As a result, the Fraction 2b elution, containing both **3** and **5**, was eventually graded and injected discretely into the prep-HPLC to avoid poor separation efficiency and loss of sample, yielding a high purity of both **3** and **5** (Figure 6b). The retention time for **3** was slightly shorter than that for **4**, based on HPLC analyses (Figure 2a), and therefore, **3** eluted from the FC was naturally precedent in comparison with **4**. As shown in Figure 6a, Peak 1 was initially identified as **3**, following the complete elution of **1** and **2**, but it was later identified as **4** by HPLC analysis. A similar result was confirmed by HPLC analysis of Fraction 1, presented in Figure 5a, while **3** was detected in Fraction 2b. This could be explained by the overload of **4** or modification of the eluotropic sequence on the MCI column.

**1** and **2**, contained in Fraction 1, were eluted as a single peak in the FC, apparently because of their closely similar retention times in the HPLC chromatogram. A mixed elution fraction was subsequently injected into the prep-HPLC for further separation, performed through eluting with 20% methanol to yield **1** and **2** with purities of 98.1% and 98.3%, respectively (Figure 6a).

**Figure 7:**
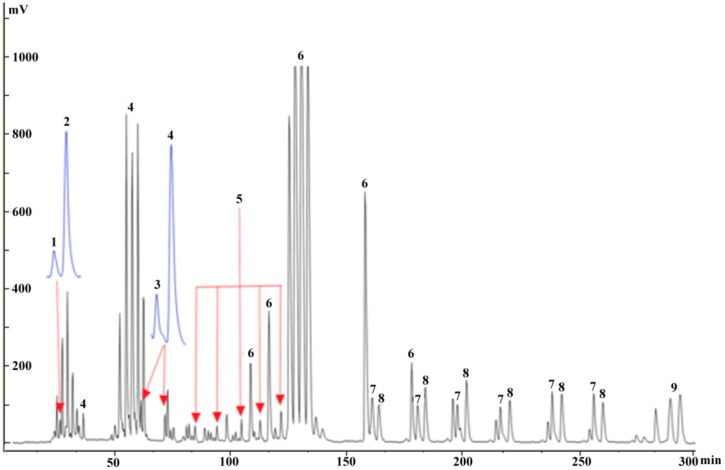
Overall separation chromatograms for the nine compounds on prep-HPLC using the coupled approach, based on offline mode separation.

As an exception, **7** and **8**, which had a relatively high content in the crude drug, were both eluted in Fraction 4, but the chromatographic peaks of these two compounds overlapped, which indicated that the mixed fraction could not be separated by continuous injection onto the prep-HPLC. Thus, the separation of **7** and **8** was repeatedly and successfully achieved offline by prep-HPLC over a Hypersil C18 column by isocratic elution with 25% methanol to yield 13.2 mg of **7** at a purity of 97.5% and 10.6 mg of **8** at a purity of 96.5% (Figure 6c). However, better resolution of **7** and **8** on prep-HPLC may possibly be achieved with a slight decrease in the ratio of the organic phase (methanol), but this might cause a longer elution time. Finally, the overall separation process for **9** by the coupled system is illustrated in Figure 7.

These results revealed that the offline mode, although requiring the suspension of the FC, appears to be useful for the separation of trace compounds. Actually, this suspension could be avoided by coupling the FC to multiple prep-HPLC via a six-way valve, as depicted in Figure 1. Furthermore, when a different type of valve is used, such as a 10-way-valve, the number of prep-HPLC attached to the FC would be increased, so more target fractions could be loaded into different prep-HPLCs through different channels, to obtain more compounds. The proposed modes (online/offline) were reliable methods for separating the compounds from *Pueraria lobata* through the combined system.

## 3. Experimental Section

### 3.1. Samples

An Isolera flash chromatography purification system from Biotage Instruments (BiotageTrading Co. Ltd., Shanghai, China) equipped with a UVdetector was used. Liquid chromatographic columns, including an MCI column (Mitsubishi Chemical Corp., Japan 60 × 22 mm i.d., average 55 μm) packed with10 g of MCI gel reversed phase adsorption resin and an ODS column (Biotage SNAP Cartridge, 60 × 22 mm i.d., average particle size of 75 μm), packed with the10 g C18 (Fuji Silysia Chemical Ltd., Osaka, Japan) stationary phase were pre-packed by our laboratory. Apreparative liquid chromatography (LC3000) instrument (Beijing Chuang Xin Tong Heng Science and Technology Co. Ltd., Beijing, China), equipped with two high-pressure solvent delivery pumps, an UV-Vis detector and a chromatography workstation, were used for further separation and purification. The preparative columns were Hypersil C18 (150 × 21.2 mm, 5 μm, Thermo Fischer Scientific, Waltham, MA, USA).The analytical high performance liquid chromatography equipment was a Shimadzu LC-20AVP system equipped with two LC-20AT solvent delivery units, an SPD-M20AVPUV-VIS photodiode array detector (DAD) system, an auto-sampler and an SCL-20AVP system controller (Shimadzu, Kyoto, Japan). Identification of the nine compounds was carried out using electrospray ionization-ion trap/mass spectra (ESI-IT/MS Agilent, Bruck Daltonics, German) and ^1^H and ^13^C nuclear magnetic resonance spectrometry (^1^H-NMR and ^13^C-NMR) (Bruker Biospin Corporation, Bremen, Germany).

### 3.2. Materials, Solvents and Chemicals

*Pueraria lobata* was purchased from Beijing RenWei Sliced Medicinal Herbs Factory (Beijing, China) and authenticated by Cheng Ming of the Institute of Chinese Materia Medica, Chinese Academy of Chinese Medical Sciences, according to its description in the Chinese Pharmacopoeia (2010 edition). Standard samples of puerarin and daidzein were purchased from the National Institute for Food and Drug Control (NIFDC, Beijing, China). All organic solvents used for crude sample preparation were of analytical grade and supplied by Beijing Chemical Factory (Beijing, China). HPLC-grade methanol (Merck KGaA, Darmstadt, Germany) was used for HPLC analyses. Deuterated solvents were supplied by J&K Scientific Ltd. (Beijing, China). Water was prepared using a Milli-Q water purification system (Millipore, Milford, MA, USA).

### 3.3. Preparation of Crude Sample from Pueraria lobata

Medicinal materials from *Pueraria lobata* (150 g) were subjected to reflux extraction with a 60% ethanol aqueous solution for 2 h. The obtained extract was then concentrated under reduced pressure in a rotary evaporator to remove the ethanol, and the crude sample extract was obtained. The crude sample extract was accurately diluted to 500 mL with water to give a crude herbal concentration of 0.3 g/mL and was filtered through a 0.45-mm PTFE filter (Iwaki Glass) to obtain the sample solution.

### 3.4. FC Coupled with Prep-HPLC Online Separation Procedure

The concentrated sample solution described above was manually loaded onto the first tandem column packed with MCI gel. A stepwise elution using different concentrations of methanol aqueous solution (20%, 25% and 30%, *v*/*v*) was conducted to obtain different fractions at a flow rate of 6.0 mL/min. All elutions were continuously monitored with an UV-Vis detector at 250 nm.

FC and prep-HPLC were connected via the six-way valve shown in Figure 1. The individual fractions were eluted and detected by the UV chromatogram from the FC. When elution fractions containing the target compounds were detected, the six-way valve was set to Position 1, and the elutions were then introduced into the injection loop of the prep-HPLC1 for the first purification. Then, the next elution fraction was introduced into the loop of prep-HPLC2 by switching the six-way valve to Position 3. Following the completion of this injection for prep-HPLC2, the six-way valve was returned to Position 1 for a second injection for prep-HPLC1. We refer to the above process as “online mode separation”, used to obtain high-content compounds. However, for low-content compounds, for which separation was much more difficult because their chromatographic peaks over lapped with those of high-content compounds during FC, the online mode may lead to a failure to achieve effective separation. In addition, the time interval between two injections may be insufficient for base line separation of the chromatographic peaks on each prep-HPLC. Hence, the offline mode (Figure 3), which required intermittent sample injection and the temporary suspension of FC, was performed during the separation procedure. That is, during the first injection of elution onto prep-HPLC1, FC was paused, and this was restarted only after the separation of the first injection was completed, to perform the second injection.

### 3.5. HPLC Analyses of Crude Sample and Identification of the Nine Compounds

The crude sample, individual FC fractions and prep-HPLC fractions were analyzed by HPLC with a DAD detector. Using the optimal isocratic elution conditions, the chromatographic peaks of the nine compounds could be separated effectively using formic acid (0.02%, *v*/*v*) and methanol (25%, *v*/*v*) aqueous solutions as the mobile phase, according to our previous study. The flow rate was set at 1.0 mL/min, with a total run time of 45 min. The characteristic chromatographic patterns were obtained by setting the detection wavelength to 250 nm, keeping the column temperature at 35 °C.

Identification of the nine compounds was achieved by HPLC-ESI-IT/MS^n^. The sample was eluted at1.0 mL/min, using an isocratic elution with 25% methanol aqueous solution, and the HPLC elution were split and introduced into the mass spectrometer and DAD detector. The detection wavelength was set in the range 190–400 nm (monitoring wavelength: 250 nm). The fragments were collected over the *m/z* 100–700 range in both the positive and negative ion modes. The mass spectrometer conditions were as follows: drying gas temperature, 300 °C; drying gas (N_2_) flow rate, 5 L/min; nebulizer, 45 psi; capillary (V), 3500; V charging, 500.

NMR spectra were obtained from the Analysis Center of Beijing University of Chemical Technology (Beijing, China) and recorded at 25 °C on a Bruker AV 600, operate data ^1^H frequency of 600.13 MHz. Chemical shifts (^1^H) were expressed in ppm and coupling constants (*J*) were reported in Hz. Compound samples were dissolved in dimethyl-sulfoxide (DMSO-*d*_6_).

### 3.6. Identification of the Nine Compounds

**Figure 8:**
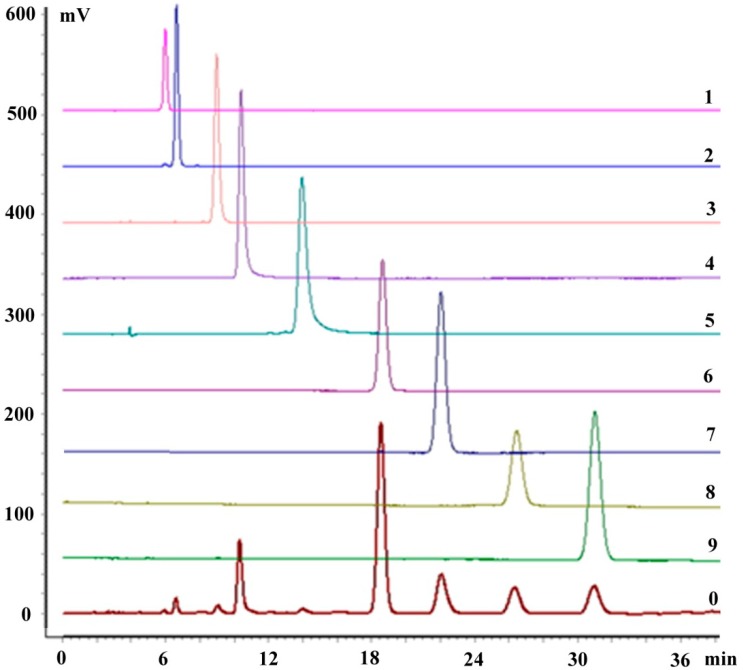
Typical HPLC analyses of the nine purified compounds (**1**–**9**) using DAD at a wavelength of 250 nm; HPLC chromatogram of the crude drugs (**0**).

The HPLC analyses shown in Figure 8 indicate that the purities of the separated compounds all exceeded 95.0%. Complete fragmentation information, based on HPLC-ESI-IT/MS^n^ analyses, is summarized in Table 1, with the structures of the nine compounds identified as 3'-hydroxypuerarin-6''-O-glucoside (**1**), puerarin-6''-O-glucoside (**2**), daidzein-4',7-O-diglucoside (**3**), 3'-hydroxypuerarin (**4**), 3'-hydroxypuerarin-6''-O-apioside (**5**), puerarin (**6**), 3'-methoxypuerarin (**7**), daidzein8-C-apiosyl(1→6)glucoside (**8**) and daidzin (**9**), respectively, and also by referring to the literature [20,21]. Compounds **4**, **6**, **7**, **8** and **9**, whose contents were relatively high, were additionally elucidated by ^1^H and ^13^C-NMR analyses.

**4** MS: *m/z* 455.2 [M+Na]^+^, indicating a molecular weight of 432 Da, which is in agreement with the molecular formula C_21_H_20_O_10_ of 3'-hydroxy puerarin. ^1^H-NMR (600 Hz, DMSO-*d*_6_) δ ppm: 8.30 (1H, s, H-2), 7.94 (1H, d, *J* = 8.8 Hz, H-5), 7.04 (1H, d, *J* = 1.9 Hz, H-2'), 7.00 (1H, d, *J* = 8.8 Hz, H-6), 6.81 (1H, dd, *J* = 8.8 Hz, 1.9 Hz, H-6'), 6.76 (1H, d, *J* = 8.2 Hz, H-5'). ^13^C-NMR (150 Hz, DMSO-*d*_6_) δ ppm: 153.1 (C-2), 123.5 (C-3), 176.3(C-4), 126.7 (C-5), 115.8 (C-6), 161.5 (C-7), 113.1 (C-8), 155.5 (C-9), 117.1 (C-10), 123.7 (C-1'), 115.8 (C-2'), 145.7 (C-3'), 145.2 (C-4'), 117.1 (C-5'), 120.2 (C-6'), 73.9 (C-1''), 71.8 (C-2''),79.2 (C-3''), 71.2(C-4'') ,82.3 (C-5''), 61.9 (C-6''). The results are consistent with those in the literature [21].

**Table 1:** MS*^n^* structural information of the nine compounds in both positive and negative ion mode.

PeakNo.	UVλ_max_(nm)	ProposedIdentification	MS^n^Data (Observed)			
			Proposed Ions (Positive Mode)	Measured Mass (*m/z*)	Proposed Ions (Negative Mode)	Measured Mass (*m/z*)
**1**	248,288	3'-hydroxy puerarin-6''-O-glucoside	[M+H]^+^	595.1		
			[M+Na]^+^	617.1	[M−H]^−^	593.0
			[M+Na−Glc]^+^	455.3	[M−H−C_4_H_8_O_4_]^−^	473.2
			[M+Na−Glc−2H_2_O]^+^	419.4	[M−H−C_4_H_8_O_4_−Glc]^−^	311.4
			[M+Na−Glc−C_4_H_8_O_4_]^+^	335.3		
**2**	248,302	Puerarin-6''-O-glucoside	[M+H]^+^	579.4		
			[M+Na]^+^	601.3	[M−H]^−^	577.3
			[M+Na−Glc]^+^	439.4	[M−H−C_4_H_8_O_4_]^−^	457.2
			[M+Na−Glc−2H_2_O]^+^	403.3	[M−H−C_4_H_8_O_4_−Glc]^−^	295.2
			[M+Na−Glc−C_4_H_8_O_4_]^+^	319.2		
**3**	250,300	Daidzein-4',7-O-diglucoside	[M+H]^+^	579.3	[M+HCOO^−^]^−^	623.7
			[M+Na]^+^	601.3	[M−H−Glc]^−^	415.2
			[M+Na−Glc]^+^	439.2	[M−H−Glc−Glc]^−^	253.2
			[M+Na−Glc−Glc]^+^	277.2		
**4**	250,310	3'-hydroxy puerarin	[M+H]^+^	433.4	[M−H]^−^	431.0
			[M+Na]^+^	455.2	[M−H−C_4_H_8_O_4_]^−^	311.0
			[M+Na−H_2_O]^+^	437.2		
			[M+Na−C_2_H_6_O_3_]^+^	377.1		
**5**	248,290	3'-hydroxy puerarin-6''-O-apioside	[M+H]^+^	565.3	[M−H]^−^	563.9
			[M+Na]^+^	587.3	[M−H−Api−Glc−CO]^−^	341.1
			[M+Na−Api]^+^	455.2	[M−H−Api−Glc−2CO]^−^	283.0
			[M+Na−Api−H_2_O]^+^	437.1		
			[M+Na−Api−C_4_H_8_O_4_]^+^	335.2		
**6**	250,310	Puerarin	[M+H]^+^	417.1	[M−H]^−^	415.2
			[M+Na]^+^	439.1	[M−H−C_4_H_8_O_4_]^−^	294.9
			[M+Na−C_4_H_8_O_4_]^+^	319.2	[M−H−C_4_H_8_O_4−_CO]^−^	267.4
			[M+Na−Glc−2H_2_O−CH_2_O]^+^	273.2		
7	250,310	3'-methoxy puerarin	[M+H]^+^	447.3	[M−H]^−^	445.5
			[M+Na]^+^	469.3	[M−H−Glc]^−^	283.3
			[M+H−H_2_O]^+^	429.3	[M−H−Glc−CH_3_]^−^	268.8
			[M+Na−H_2_O−C_2_H_6_O_3_]^+^	373.3		
			[M+Na−H_2_O−C_2_H_6_O_3−_CH_3_]^+^	357.2		
**8**	249,306	Daidzein 8-*C*-apiosyl (1→6)glucoside	[M+H]^+^	549.3	[M−H]^−^	547.4
			[M+Na]^+^	571.3	[M−H−Api−Glc−CO]^−^	325.0
			[M+Na−Api]^+^	439.2	[M−H−Api−Glc−2CO]^−^	267.1
			[M+Na−Api−Glc]^+^	277.1		
**9**	250,310	Daidzin	[M+H]^+^	417.1	[M−H]^−^	415.0
			[M+Na]^+^	439.1	[M+HCOO^−^]^−^	461.0
			[M+Na−Glc]^+^	277.1	[M−H−Glc]^−^	253.0
			[M+H−Glc]^+^	255.1		

**6** MS: *m/z* 417.2 [M+H]^+^, consistent with a molecular formula of C_22_H_22_O_9_. ^1^H-NMR (600 MHz, DMSO-*d*_6_) δ ppm: 8.36 (1H,s, H-2), 7.94 (1H,d, *J* = 8.8 Hz, H-5), 7.40 (2H,d, *J =* 7.0 Hz, H-3',5'), 7.00 (1H,d, *J* = 8.6 Hz, H-6), 6.81 (2H,d, *J* = 7.1 Hz, H-2',6'); ^13^C-NMR (150 MHz, DMSO-*d*_6_) δ ppm: 153.1 (C-2), 123.5 (C-3), 175.4 (C-4), 126.7 (C-5), 115.4 (C-6), 161.5 (C-7), 113.1 (C-8), 156.5 (C-9), 117.3 (C-10), 123.5 (C-1'), 130.5 (C-2'), 115.4 (C-3'), 157.6 (C-4'), 115.4 (C-5'), 130.5 (C-6'), 73.8 (C-1''), 71.2 (C-2''), 79.2 (C-3''), 70.8 (C-4''), 82.3 (C-5''), 61.9 (C-6'').These findings are consistent with the literature data [21].

**7** MS: *m/z* 447.3 [M+Na]^+^, indicating a molecular weight of 424 Da, which is consistent with a molecular formula of C_22_H_22_O_10_. ^1^H-NMR (600 Hz, DMSO-*d*_6_) δ ppm: 8.41 (1H,s,H-2), 7.94 (1H,d, *J* = 8.8 Hz, H-5), 7.17 (1H, d, *J* = 1.8 Hz, H-2'), 7.05 (1H, d, *J* = 8.8 Hz, H-6), 7.00 (1H, dd, *J* = 8.8 Hz, 1.8 Hz, H-6'), 6.81 (1H, d, *J* = 8.8 Hz, H-5'), 4.82 (1H, d, *J* = 9.7 Hz), 3.81 (3H, s, -OMe). ^13^C-NMR (150 Hz, DMSO-*d*_6_) δ ppm: 153.4 (C-2), 123.5 (C-3), 175.3 (C-4), 126.7 (C-5), 115.6 (C-6), 161.8 (C-7), 113.1 (C-8), 156.6 (C-9), 117.2 (C-10), 123.5 (C-1'), 113.5 (C-2'), 146.9 (C-3'), 147.7 (C-4'), 115.6 (C-5'), 122.0 (C-6'), 73.9 (C-1''), 71.2 (C-2''), 79.3 (C-3''), 71.0 (C-4''), 82.4 (C-5''), 61.9 (C-6''), 56.1 (-OMe). The results are consistent with those in literature data [21].

**8** MS: *m/z* 549.3 [M+H]^+^, indicating a molecular weight of 548 Da, consistent with the molecular formula C_26_H_28_O_13_ of daidzein8-C-apiosyl(1→6) glucoside. ^1^H-NMR (600 Hz, DMSO-*d*_6_) δ ppm: 8.33 (1H, s, H-2), 7.93 (1H,d, *J* = 8.8 Hz, H-5), 7.40 (2H,d, *J* = 8.5 Hz, H-2', H-6'), 6.99 (1H, d, *J* = 8.5 Hz, H-6), 6.89 (2H, d, *J* = 8.5 Hz, H-3', H-5'), 4.80 (1H, d, *J* = 9.7 Hz), 4.78 (1H, d, *J* = 3.0 Hz). ^13^C-NMR (150 Hz, DMSO-*d*_6_) δ ppm: 153.1 (C-2), 123.0 (C-3), 175.4 (C-4), 126.7 (C-5), 115.4 (C-6), 161.9 (C-7), 112.9 (C-8), 156.6 (C-9), 117.2 (C-10), 123.5 (C-1'), 130.5 (C-2'), 115.4 (C-3'), 157.6 (C-4'), 115.4 (C-5'), 130.5 (C-6'), 73.9 (C-1''), 71.0 (C-2''), 79.2 (C-3''), 70.1 (C-4''), 80.5 (C-5''), 68.8 (C-6''), 109.5 (C-1'''), 76.1 (C-2'''), 79.1 (C-3'''), 73.7 (C-4'''), 63.4 (C-5'''). The results are consistent with those in literature data [21].

**9** MS: *m/z* 415.0 [M−H]^−^, indicating a molecular weight of 416 Da, consistent with the molecular formula C_26_H_28_O_13_ of daidzein. ^1^H-NMR (600 MHz, DMSO-*d*_6_) δ ppm: 9.56 (1H, s, 4'-OH), 8.40 (1H, s, H-2), 8.05 (1H, d, *J* = 8.9 Hz, H-5), 7.41 (2H, d, *J* = 8.6 Hz, H-2',6'), 7.24 (1H, d, *J* = 2.2 Hz, H-8), 7.15 (1H, dd, *J* = 8.8, 2.2 Hz, H-6), 6.82 (2H, d, *J* = 8.6 Hz, H-3', 5'), 5.11 (1H, d, *J* = 7.4 Hz, H-8). ^13^C-NMR (150 MHz, DMSO-*d*_6_) δ ppm: 153.8 (C-2), 122.8 (C-3), 175.2 (C-4), 127.4 (C-5), 116.1 (C-6), 161.9 (C-7), 103.8 (C-8), 157.7 (C-9), 118.9 (C-10), 124.2 (C-1'), 130.6 (C-2',6'), 115.5 (C-3',5'), 157.5 (C-4'), 100.4 (C-1''), 73.6 (C-2''), 77.0 (C-3''), 70.1 (C-4''), 77.7 (C-5''), 61.1 (C-6''). These data are in agreement with those reported in the literature [16].

## 4. Conclusions

This pioneering research has developed an ovelap proach combining FC and prep-HPLC for the efficient isolation and purification of nine isoflavonoid compounds from *Pueraria lobata*. In this study, different elutions were fractionated by FC and sequentially loaded onto prep-HPLC columns for further purification. We proposed two separation modes, an online mode that successfully separated three (high-content) principal compounds (3'-hydroxypuerarin (**4**), puerarin (**6**) and daidzin (**9**)) and an offline mode. In the offline mode, 3'-hydroxypuerarin-6''-*O*-glucoside (**11**), puerarin-6''-*O*-glucoside (**2**), daidzein-4',7-*O*-diglucoside (**3**),3'-hydroxypuerarin-6''-*O*-apioside (**5**),3'-methoxypuerarin (**7**) and daidzein8-C-apiosyl (1→6) glucoside (**8**) were separated. The offline mode requires the suspension of FC, but it was advantageous for the isolation of trace (relatively low-content) constituents in this experiment. The purities of the nine target compounds all exceeded 95.0% using the combined system. In addition, the coupled system substantially reduces solvent consumption and process time compared with traditional methods, while yielding the same quantity of high content compounds from *Pueraria lobata*.

Our study demonstrates that the combined approach is superior to conventional methods, because it increases peak capacity and is rapid, convenient and economical. Compared with conventional chromatographic methods, it saves time and is very suitable for the separation of natural products, which are often complex. Additionally, fractions isolated by FC can be directly transferred and injected onto prep-HPLC columns, which avoids their reversible absorption of the sample on the solid phase. Overall, the method described here is expected to be useful for the simultaneous separation of both high-content and low-content compounds from natural products and to offer a promising alternative to routine separation technologies for bioactive constituents.

## Supplementary Materials

Supplementary materials can be accessed at: http://www.mdpi.com/1420-3049/20/03/4238/s1.
