# Supplementary material for: A Novel and Effective Chromatographic Approach to the Separation of Isoflavone Derivatives from Pueraria lobata
Source: Molecules. 2015 Mar 5;20(3):4238–53. doi: 10.3390/molecules20034238 (PMC6272553; doi:10.3390/molecules20034238)
Supplement: Supplementary file 1 [file molecules-20-04238-s001.pdf]

Supplementary Materials

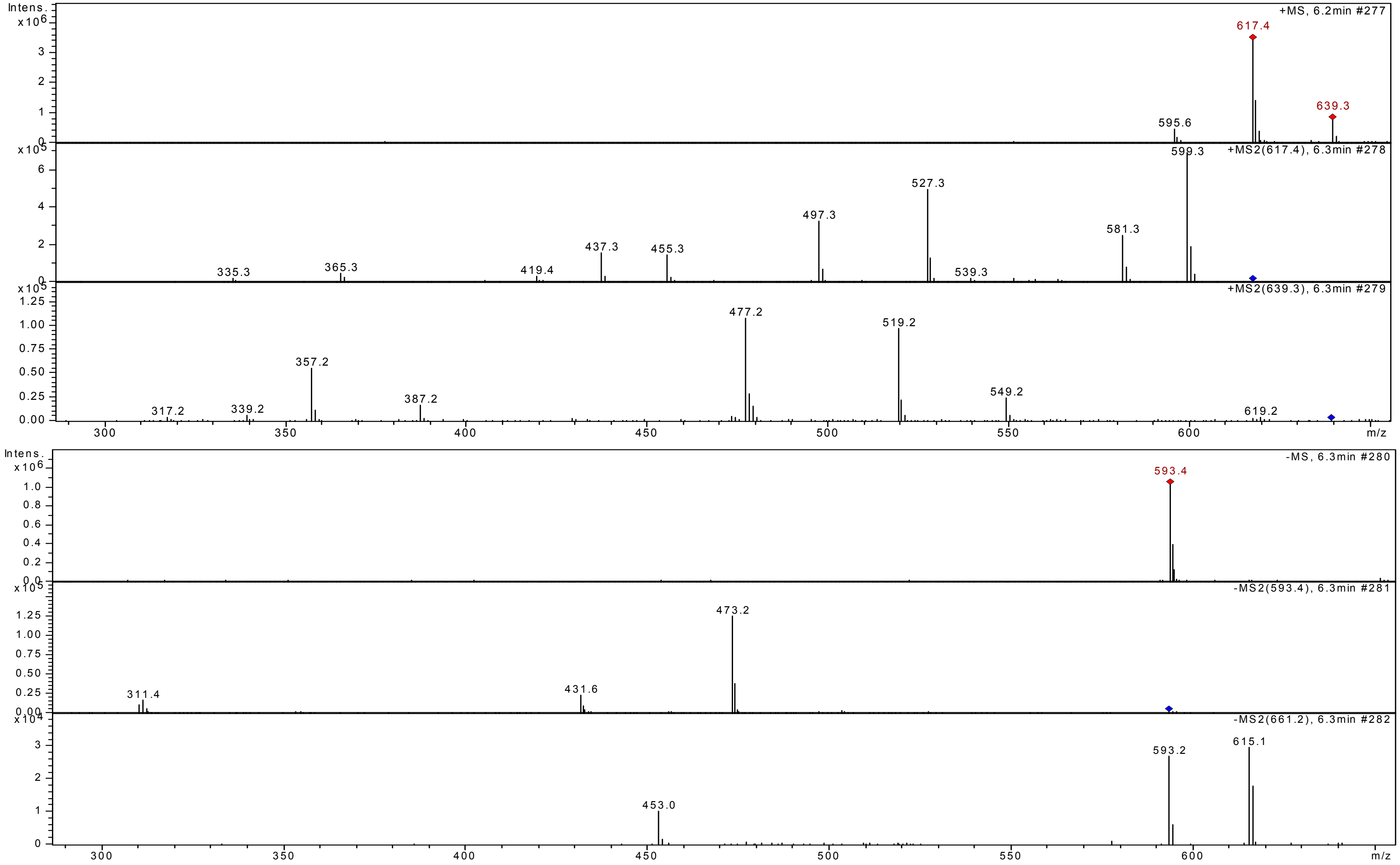

Figure S1. 1-MS raw data.

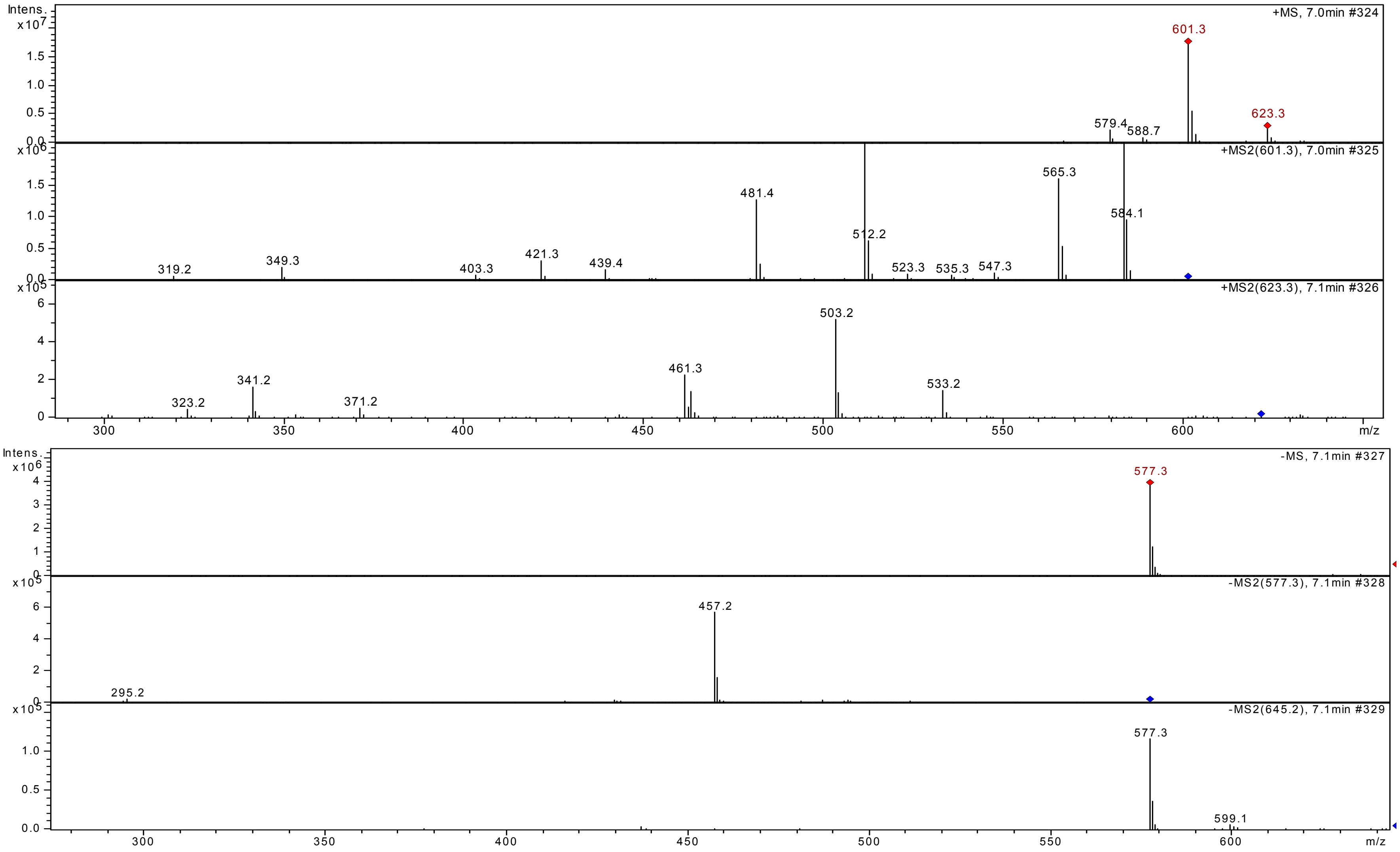

Figure S2. 2-MS raw data.

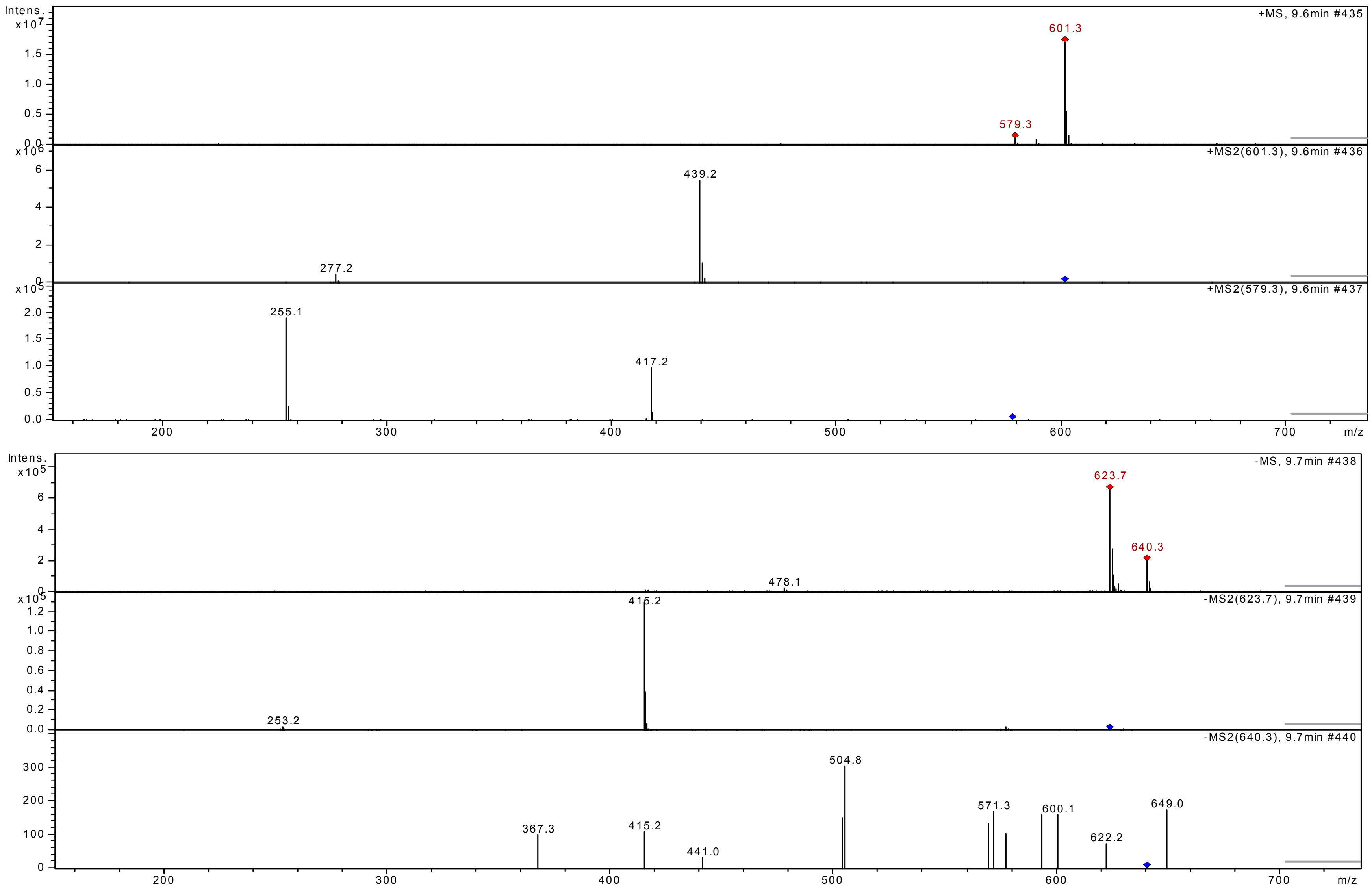

Figure S3. 3-MS raw data.

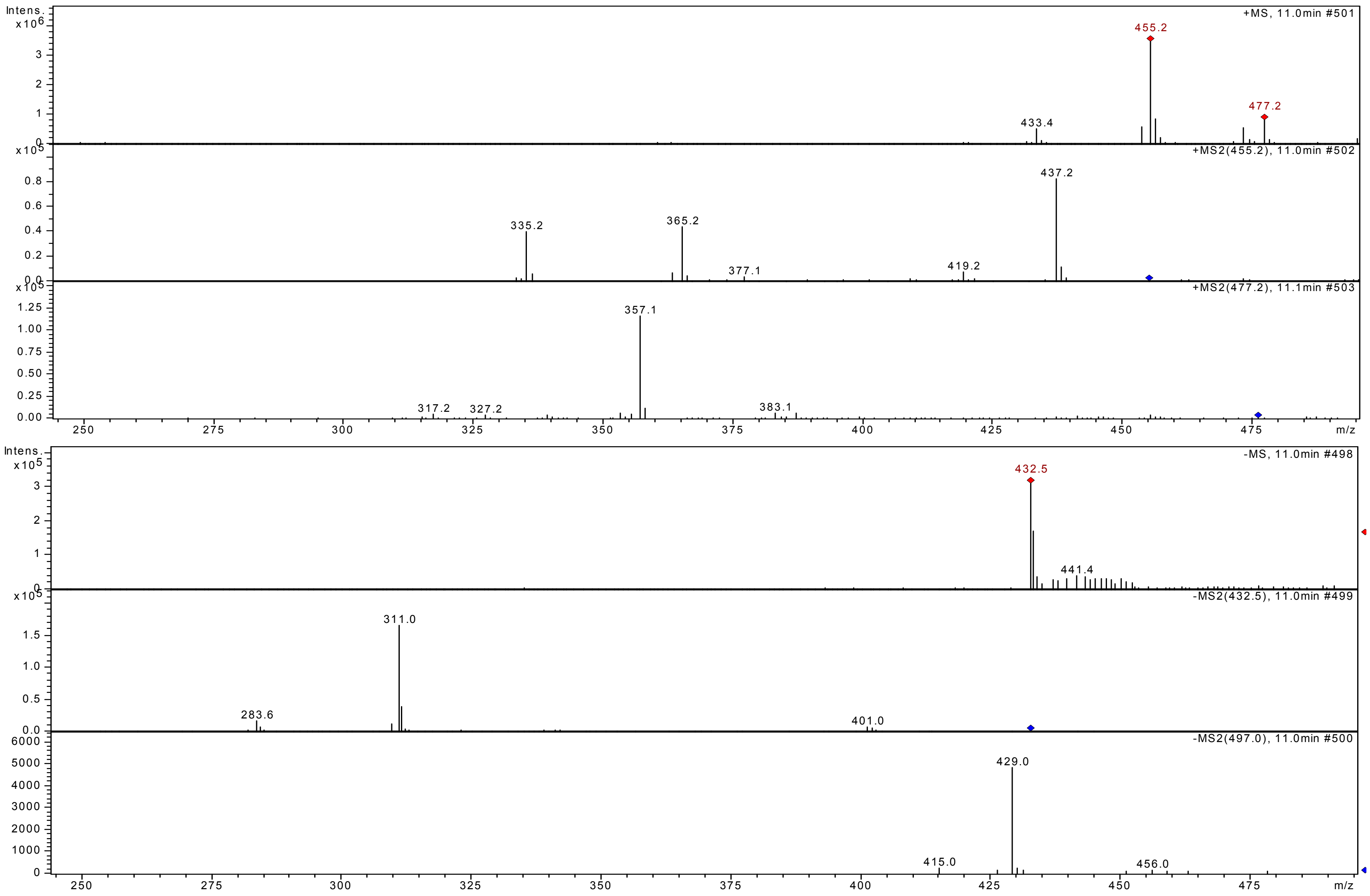

Figure S4. 4-MS raw data.

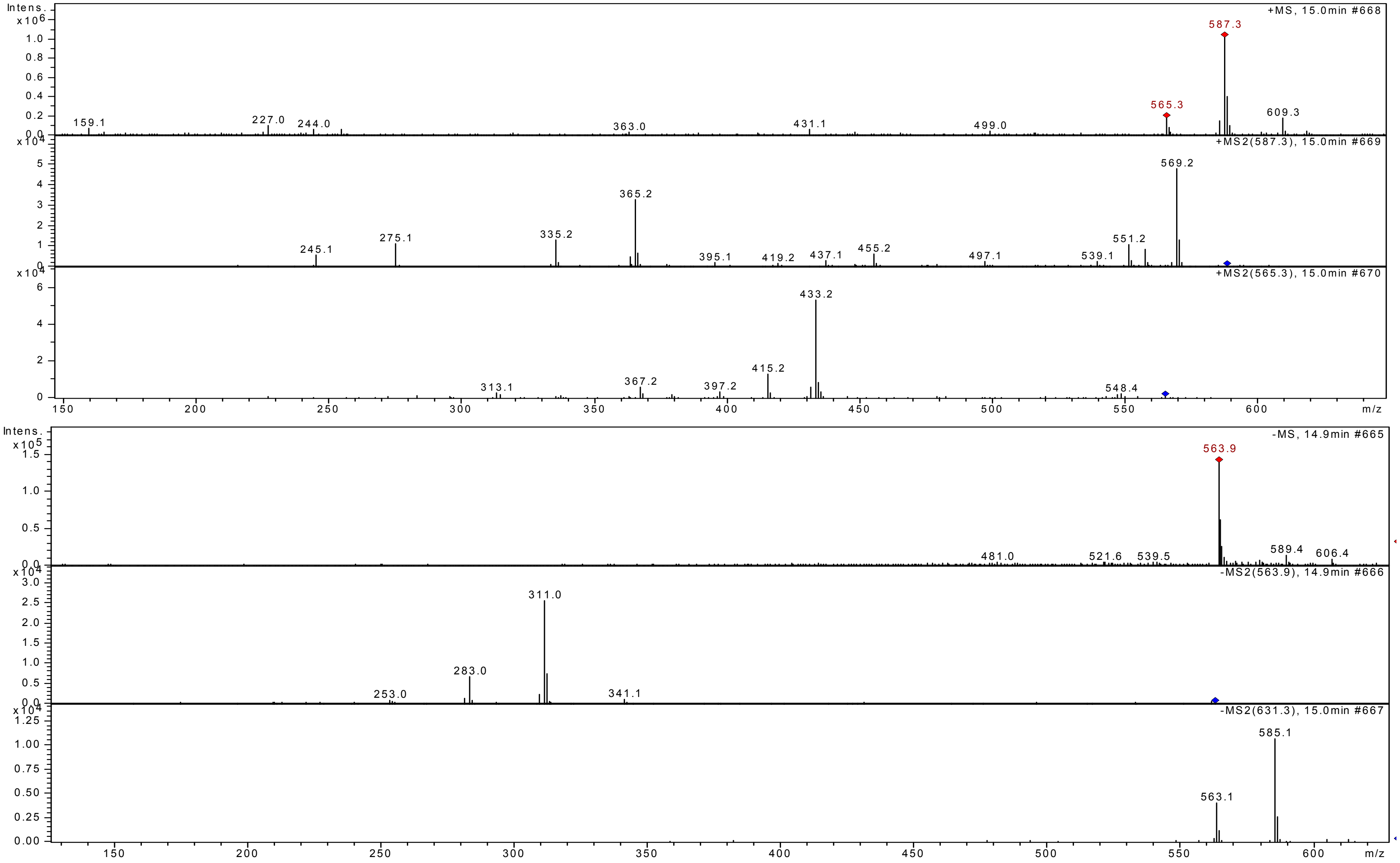

Figure S5. 5-MS raw data.

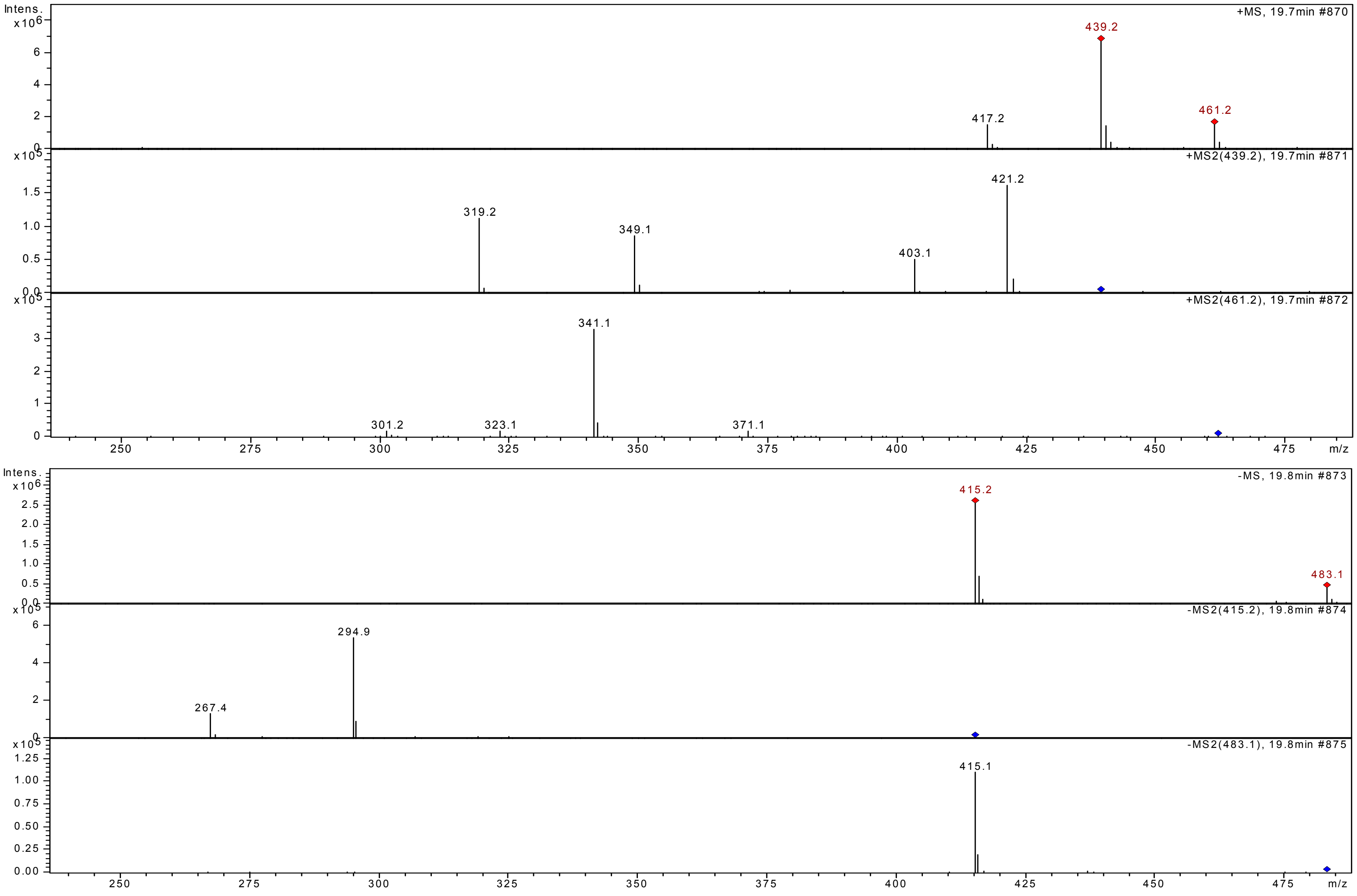

Figure S6. 6-MS raw data.

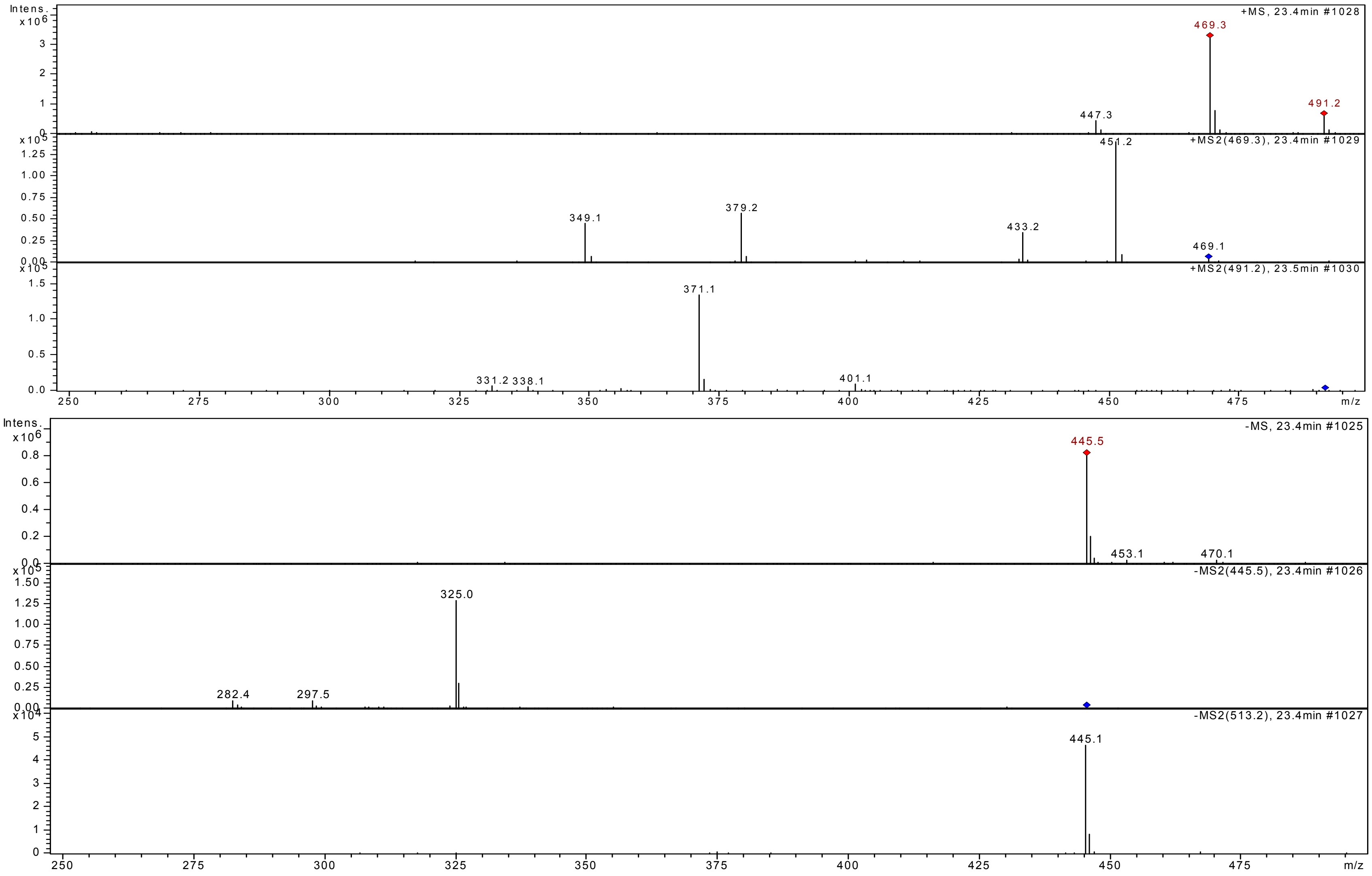

Figure S7. 7-MS raw data.

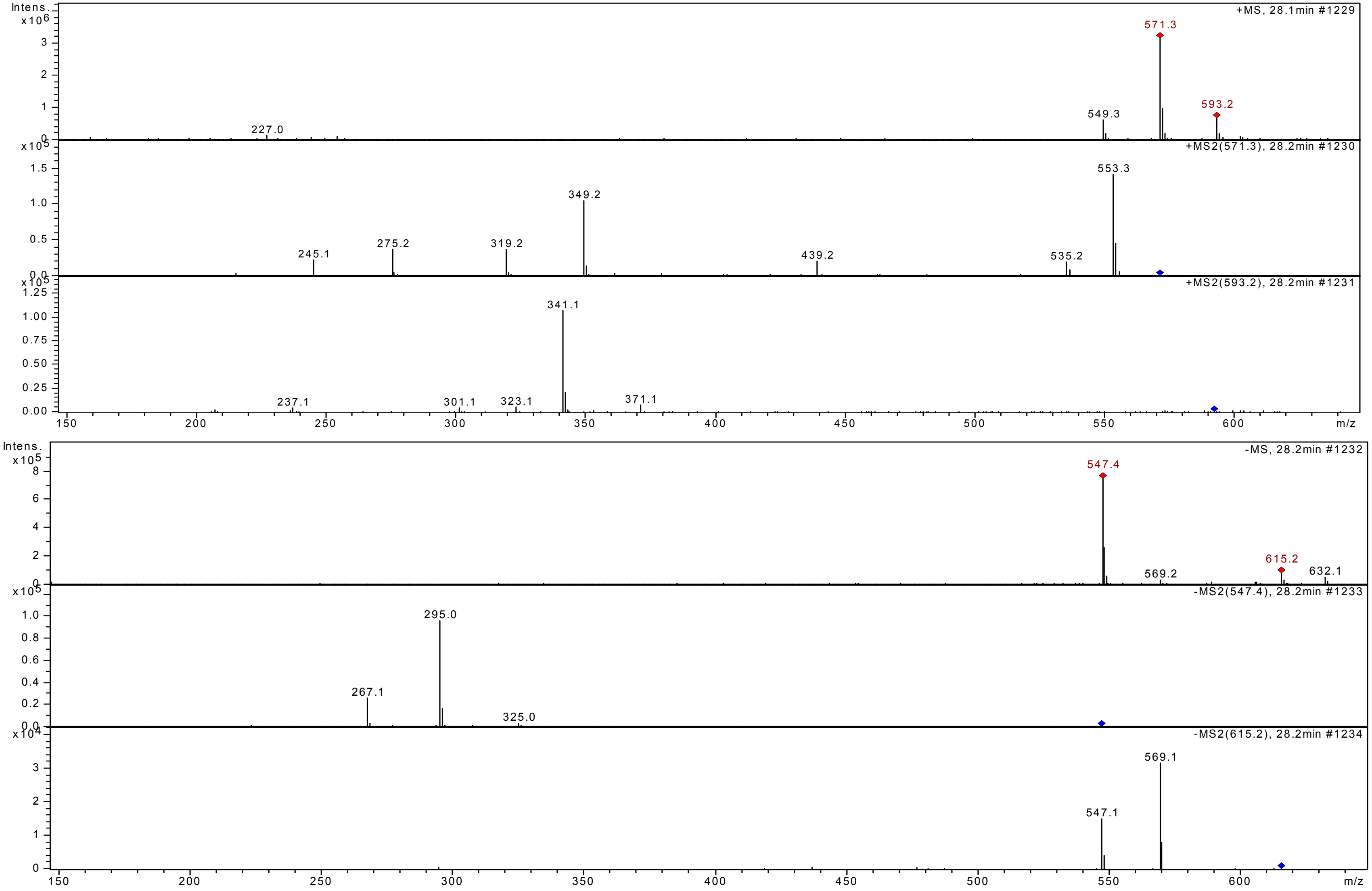

Figure S8. 8-MS raw data.

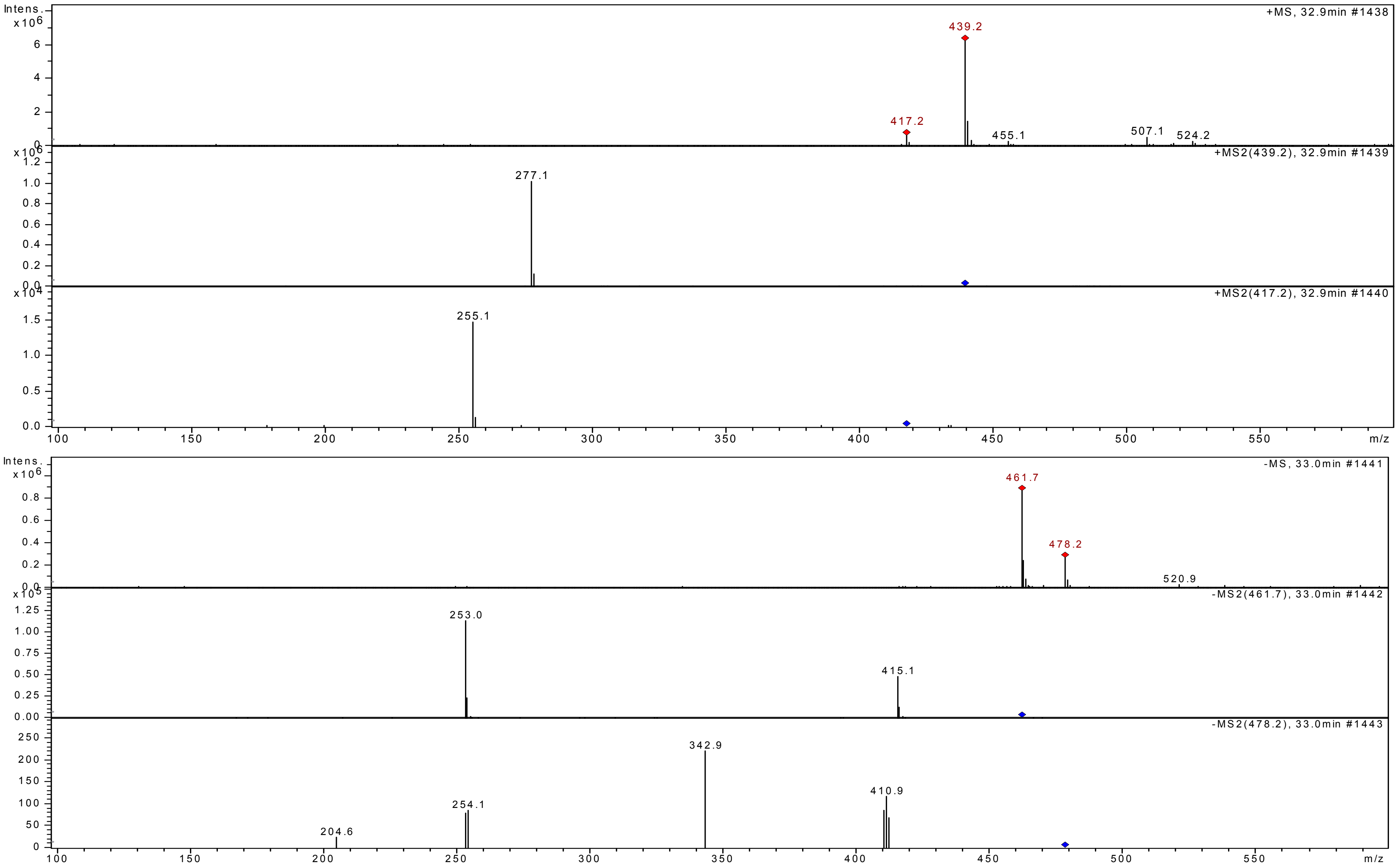

Figure S9. 9-MS raw data.

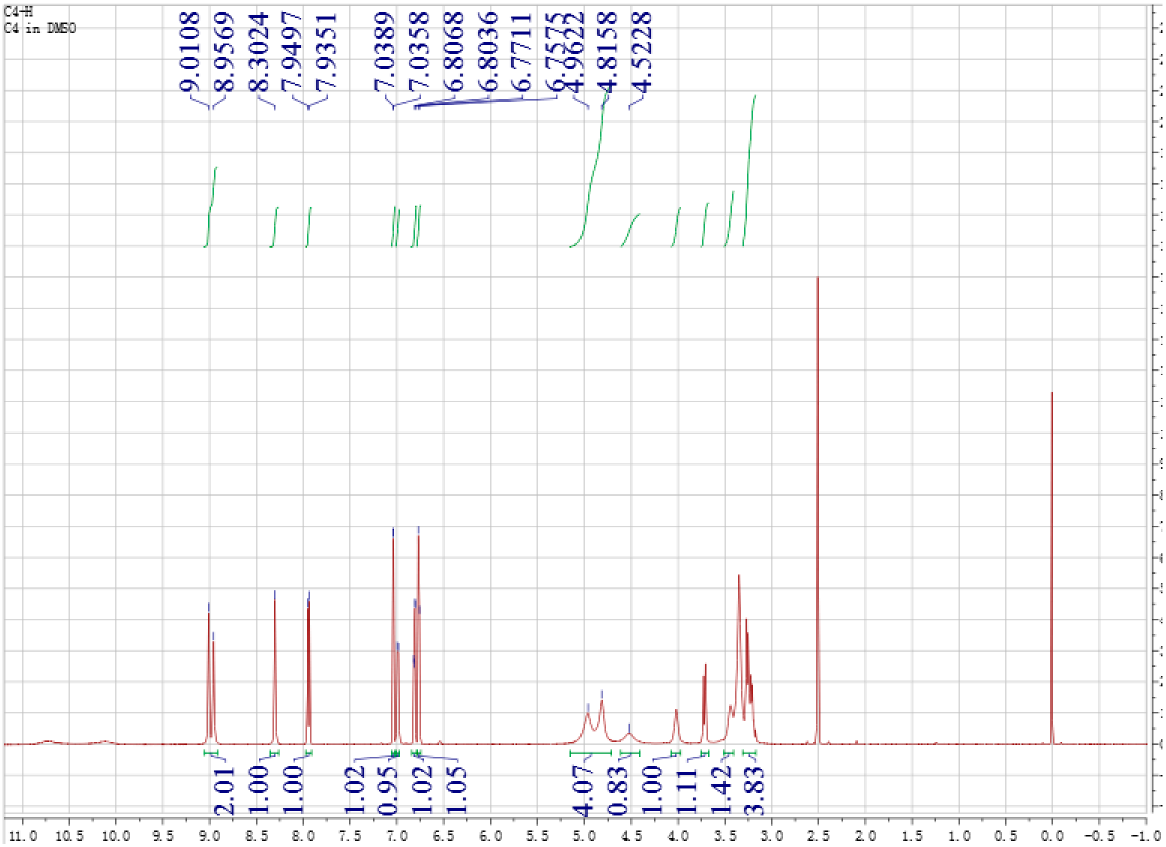

Figure S10. 4-^{1}H-NMR raw data.

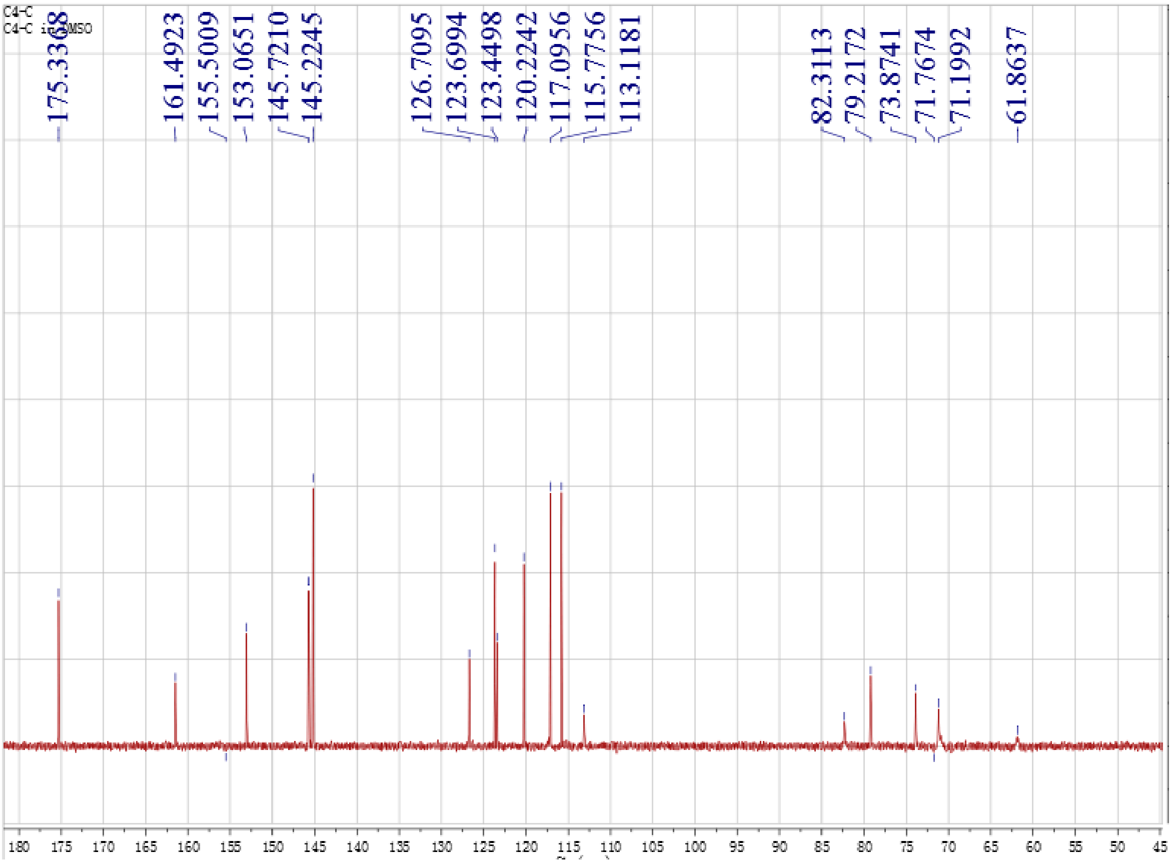

Figure S11. 4-^{13}C-NMR raw data.

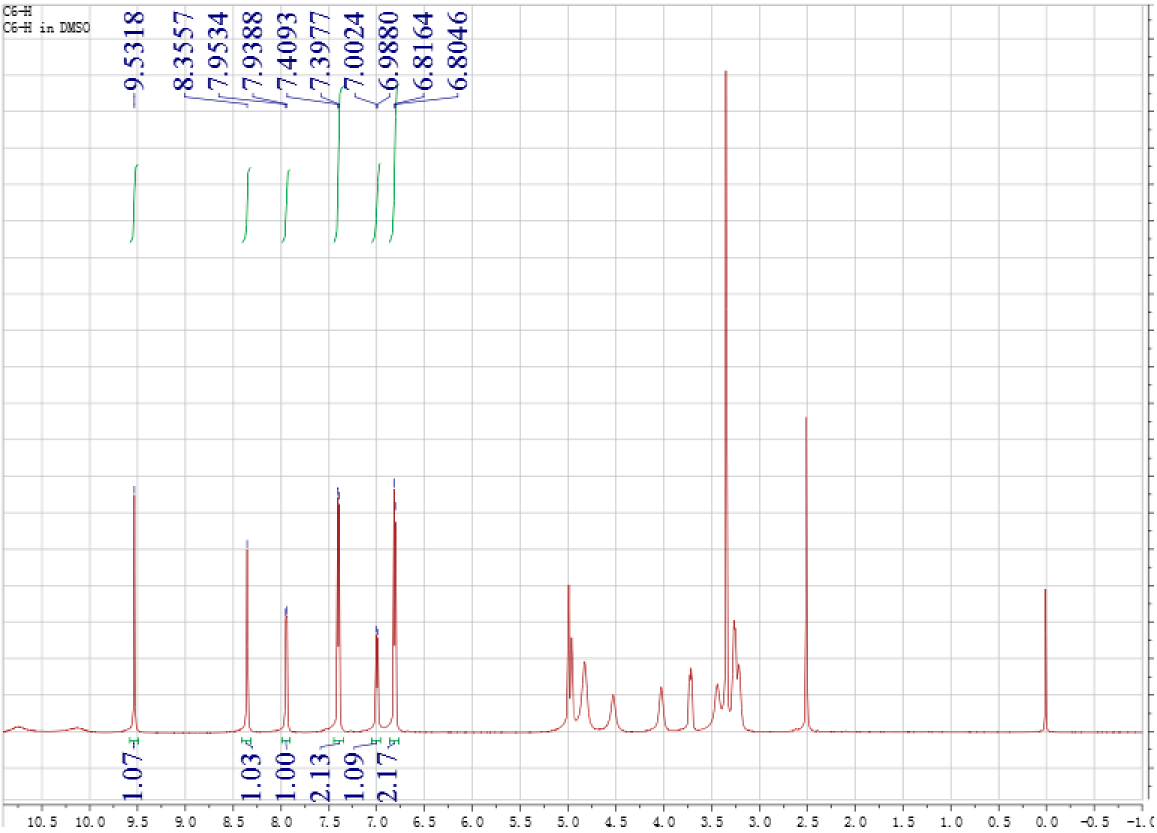

Figure S12. 6-^{1}H-NMR raw data.

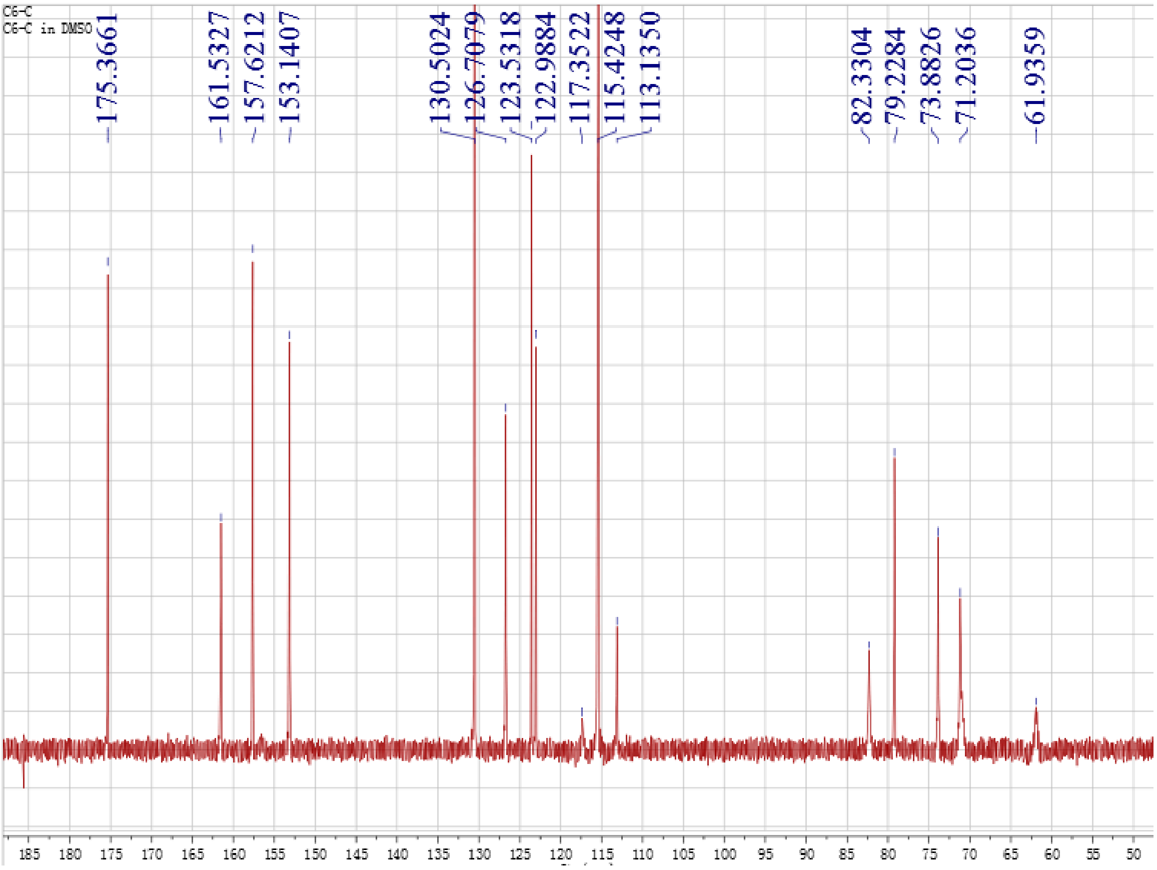

Figure S13. 6-^{13}C-NMR raw data.

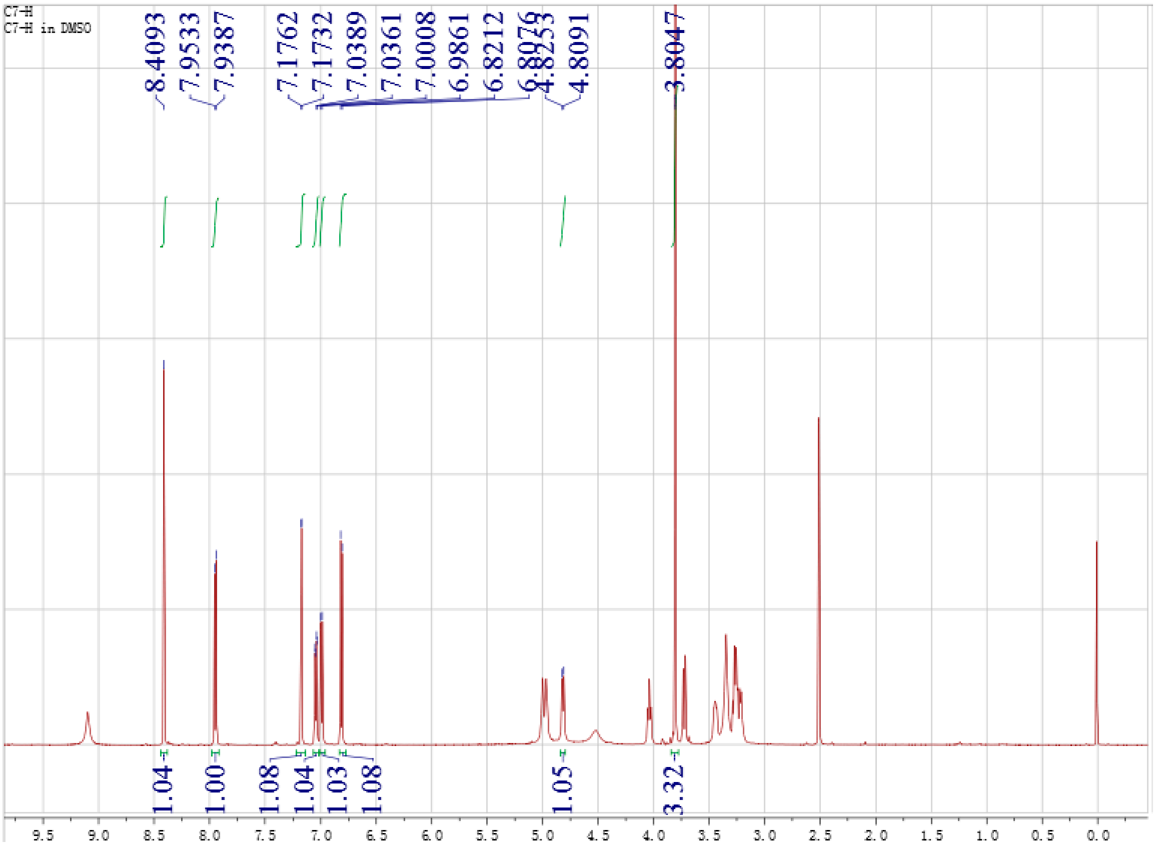

Figure S14. 7-^{1}H-NMR raw data.

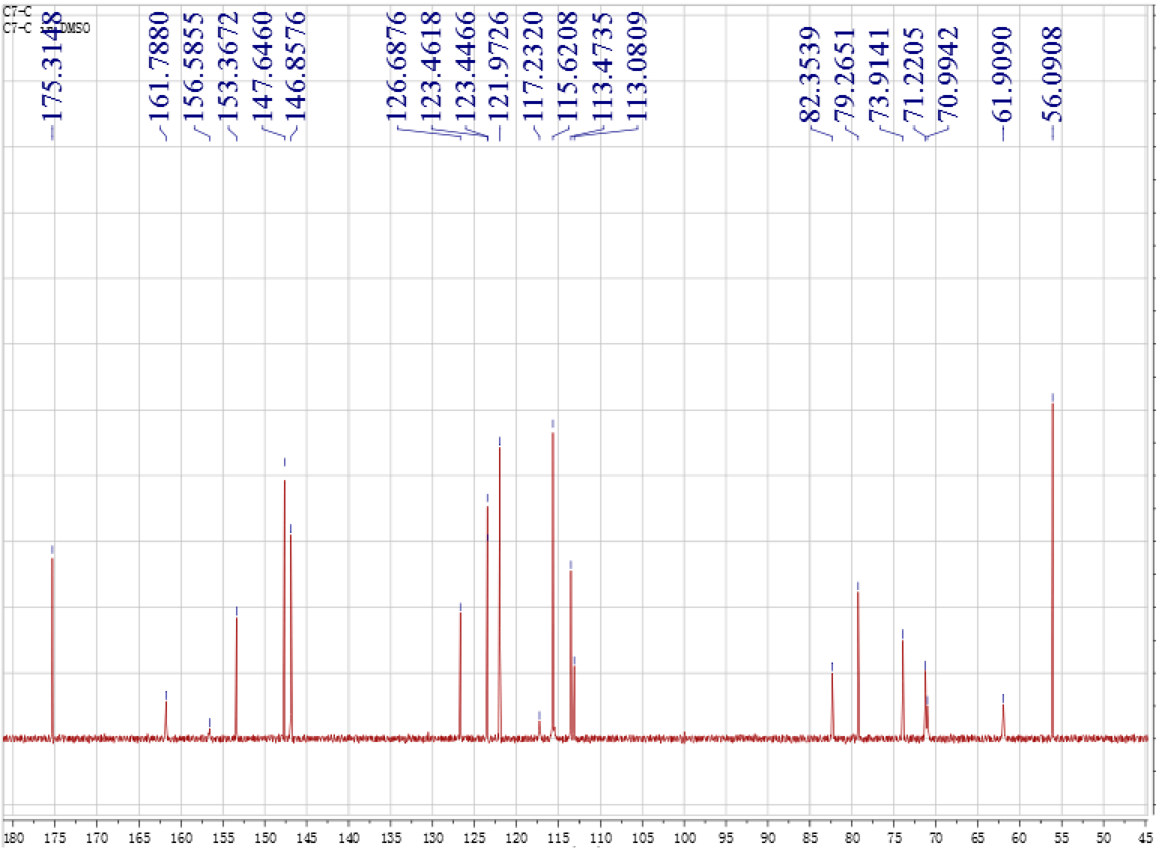

Figure S15. 7-^{13}C-NMR raw data.

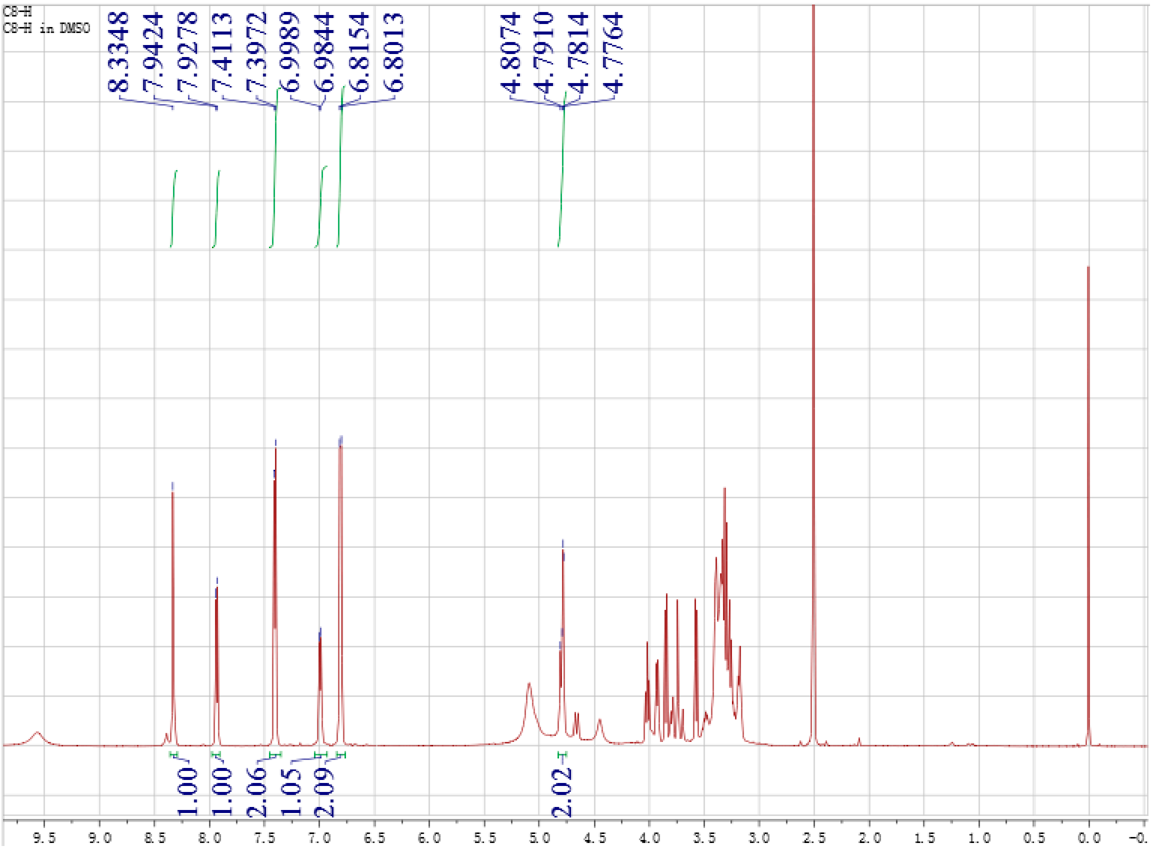

Figure S16. 8-^{1}H-NMR raw data.

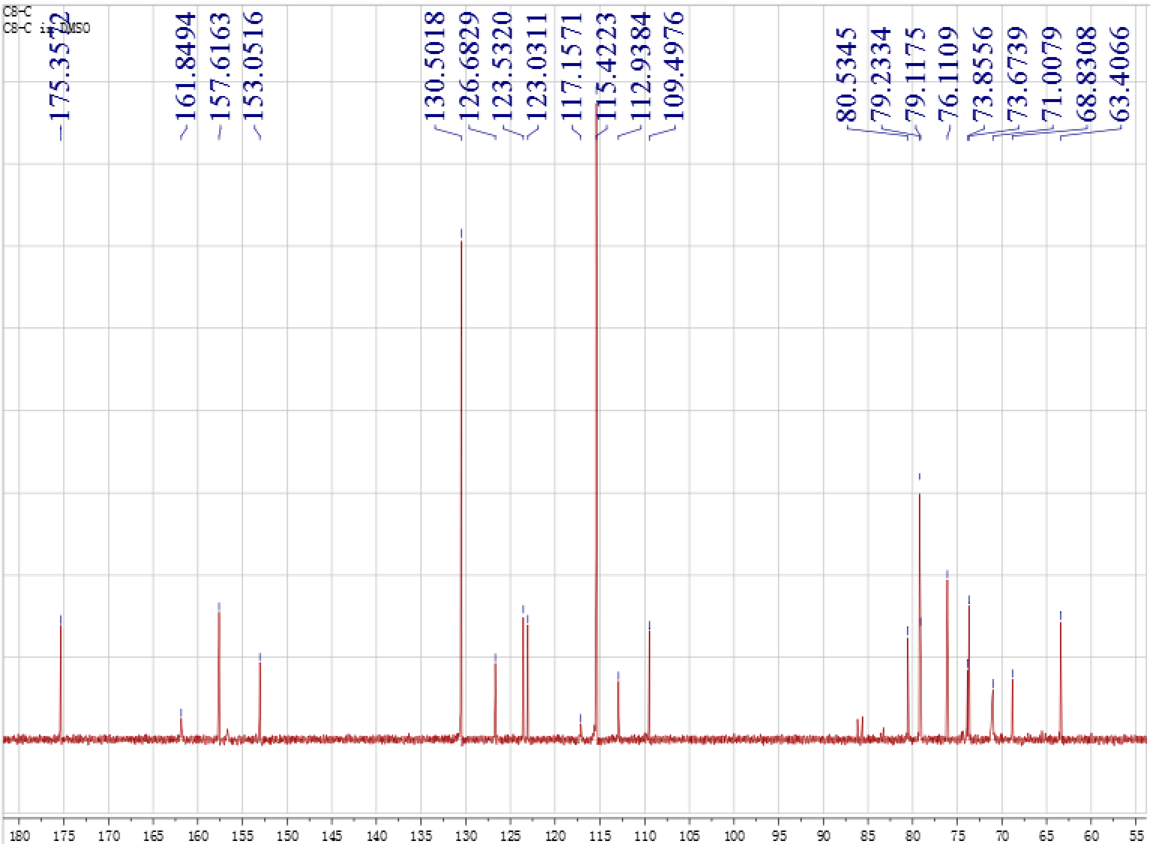

Figure S17. 8-^{13}C-NMR raw data.

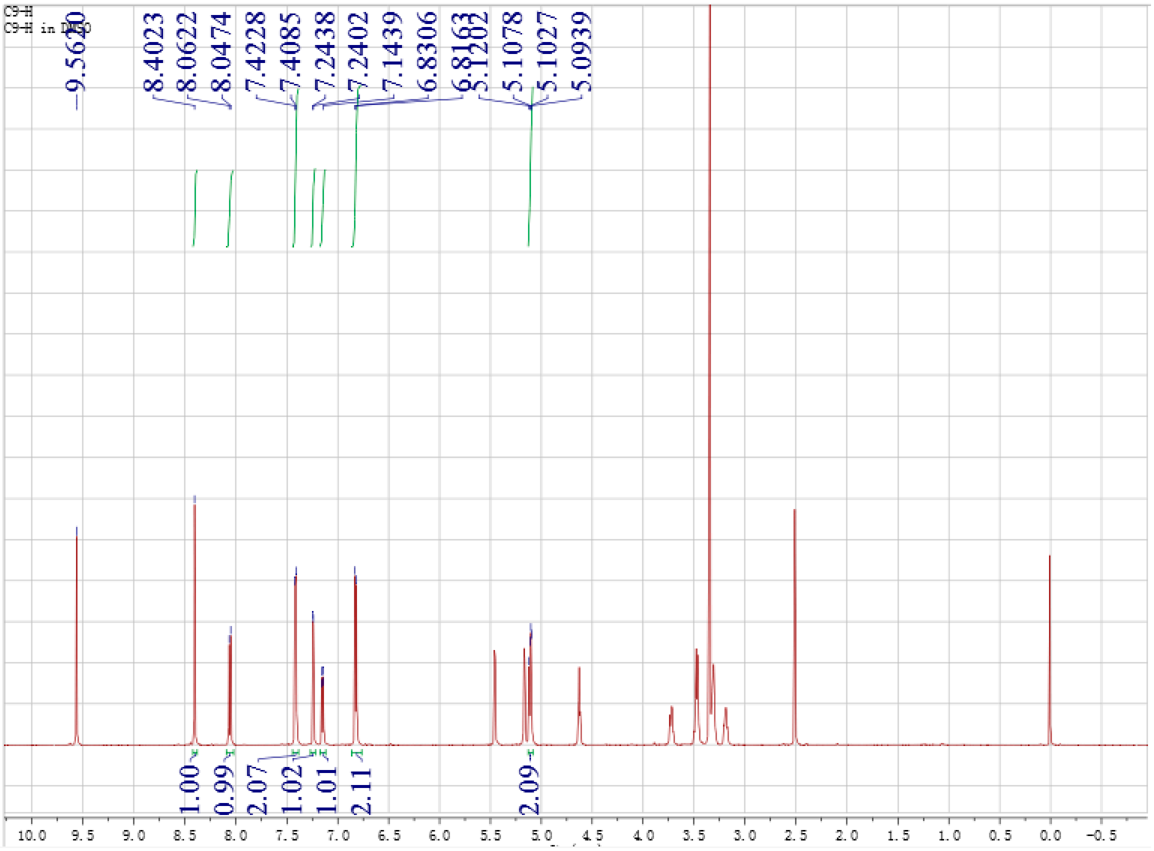

Figure S18. 9-^{1}H-NMR raw data.

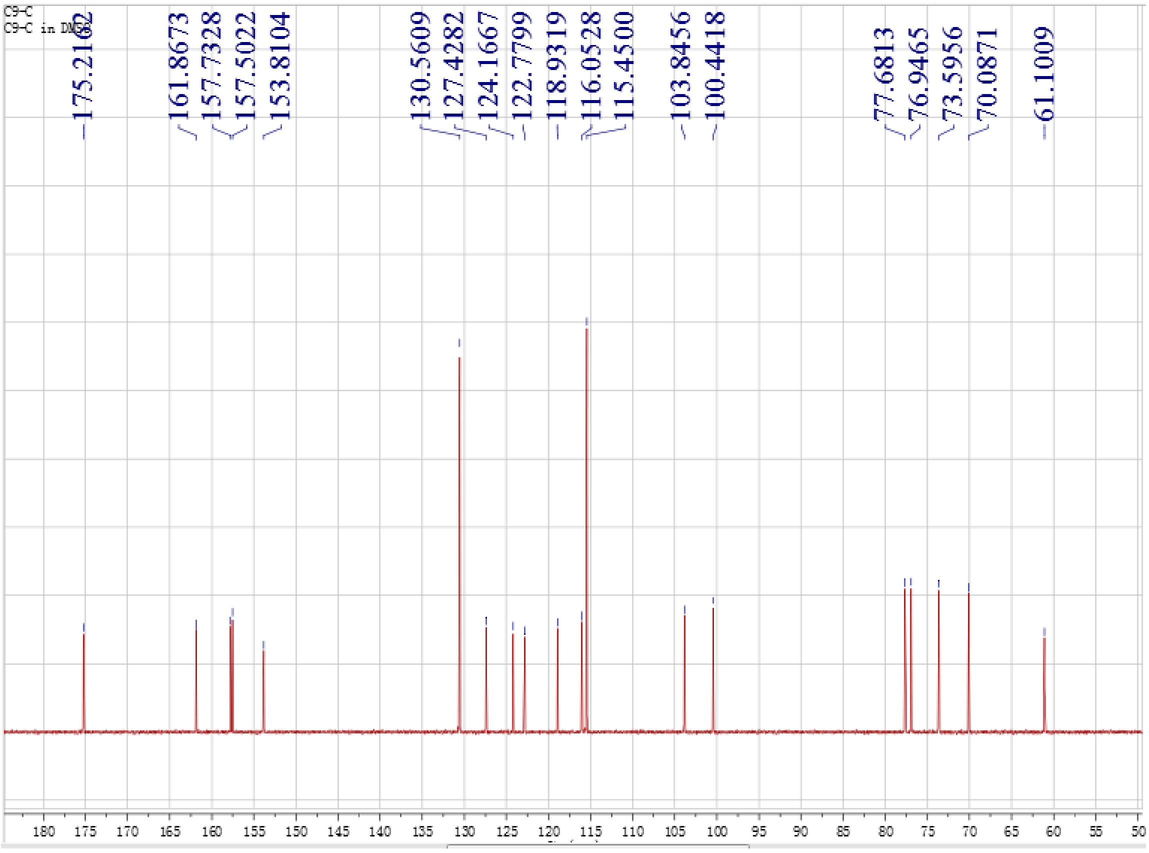

Figure S19. 9-^{13}C-NMR raw data.
